# The biological basis of Blood-Heat syndrome in children with Henoch-Schonlein purpura nephritis: a multidimensional analysis based on clinical proteomics and an animal model

**DOI:** 10.3389/fphar.2026.1778919

**Published:** 2026-04-10

**Authors:** Shuang Xu, Yan Xu, Yuefeng Bi, Ying Ding, Xia Zhang, Leying Xi, Xianqing Ren

**Affiliations:** 1 Pediatric Hospital of the First Affiliated Hospital of Henan University of Chinese Medicine, Zhengzhou, Henan, China; 2 School of Pediatrics, Henan University of Chinese Medicine, Zhengzhou, Henan, China; 3 The First Affiliated Hospital of Henan University of Chinese Medicine, Zhengzhou, Henan, China; 4 School of Pharmacy, Zhengzhou University, Zhengzhou, Henan, China

**Keywords:** biomarkers, Blood-Heat syndrome, inflammation, proteomics, sphingolipid signaling pathway

## Abstract

**Objective:**

Blood-Heat syndrome is a core syndrome of Traditional Chinese Medicine (TCM) in Henoch-Schönlein purpura nephritis (HSPN), yet its biological basis remains unclear. This study aimed to systematically elucidate the scientific basis of Blood-Heat syndrome within the context of HSPN and to identify its objective biomarkers using a multidimensional biological approach.

**Methods:**

In the clinical research part, we divided it into a discovery cohort and a validation cohort. The discovery cohort employed Data-Independent Acquisition (DIA) proteomics technology to analyze serum samples from HSPN patients with Blood-Heat syndrome (n = 15), those without Blood-Heat syndrome (non-Blood-Heat, n = 30), and healthy controls (n = 30). The findings were then validated through ELISA in both the discovery cohort and an independent validation cohort (n = 30 for blood heat syndrome, n = 30 for non-blood heat syndrome). In the basic research component, we established a rat model combining HSPN with Blood-Heat syndrome to replicate the clinical findings.

**Results:**

Proteomic analysis identified 87 specific differentially expressed proteins (DEPs) associated with Blood-Heat syndrome. Kyoto Encyclopedia of Genes and Genomes (KEGG) analysis revealed significant enrichment in the sphingolipid signaling pathway (*P* = 0.02). We further identified a panel of nine core biomarkers (AHSG, HRG, KNG1, HP, AZGP1, PTX3, MAPK1, A1BG, and COL1A1), which demonstrated excellent diagnostic performance in distinguishing between healthy control group and blood-heat syndrome, as well as between blood-heat syndrome and non-blood-heat syndrome (with AUC values all ≥0.7). ELISA validation showed that, compared to the healthy control group and non-Blood-Heat group, the levels of AHSG, HRG, and KNG1 were significantly downregulated in the Blood-Heat group, while the other six markers were significantly upregulated (*P* < 0.01 for all). This trend was fully replicated in the HSPN Blood-Heat syndrome rat model.

**Conclusion:**

Based on multidimensional evidence from clinical proteomics and animal model replication, this study suggests that Blood-Heat syndrome in the context of HSPN has a reproducible molecular phenotype. The functional enrichment of its differential proteins involves the sphingolipid signaling pathway, accompanied by an enhanced inflammatory background represented by ERK2 upregulation. Based on these findings, we propose a core scientific hypothesis of “Blood-Heat-related stress—sphingolipid signaling-associated alterations—ERK2-mediated inflammatory amplification,” providing a direction for future mechanistic validation and targeted intervention research.

## 1 Introduction

The syndrome of Traditional Chinese Medicine (TCM), or *Zheng*, is a cornerstone of the TCM theoretical system and the foundation for clinical “syndrome differentiation and treatment” (*Bian Zheng Lun Zhi*). However, the diagnosis of TCM syndromes has long relied on the macroscopic and comprehensive judgment of physicians through “inspection, auscultation, olfaction, inquiry, and palpation,” a process whose subjectivity and conceptual abstraction have hindered its full understanding and acceptance within the modern biomedical system. Elucidating the objective biological basis of TCM syndromes is a critical scientific challenge for the modernization of TCM and an essential step toward the deep integration of Chinese and Western medicine (Li et al., 2025).

Among the numerous TCM syndromes, Blood-Heat syndrome is a core pathological state that runs through various inflammatory and hemorrhagic diseases. Its fundamental pathogenesis involves heat evil invading the blood aspect, leading to accelerated blood circulation or “heat forcing the blood to move recklessly.” To find a verifiable modern biological anchor for this abstract concept, selecting an appropriate disease model is crucial. Henoch-Schönlein Purpura Nephritis (HSPN) provides an important research vehicle for this purpose. The clinical and pathological classification of HSPN is closely related to prognosis. Although renal biopsy is a key diagnostic tool, its invasiveness and limited repeatability restrict its feasibility for dynamic assessment in children, thus creating an urgent need for non-invasive biomarker systems based on body fluid omics (Zhou et al., 2025; Fang et al., 2020). Phenotypically, the hallmark clinical manifestation of HSPN, cutaneous purpura, is highly consistent with the classic pathogenesis of Blood-Heat syndrome, “heat forcing the blood to move, causing blood to overflow from the vessels.” Previous clinical studies have indicated that Blood-Heat syndrome is a common clinical pattern in HSPN (Ding et al., 2024; Liu et al., 2011). Therefore, using HSPN as the “disease” carrier to study the essence of “Blood-Heat syndrome” has a clear clinical phenotype basis and translational value.

From a pathophysiological perspective, HSPN is an IgA vasculitis-associated nephritis. Its renal damage process can be driven by immune complex-related inflammation and is coupled with innate immune effector mechanisms such as the complement system (Scientific Committee of the China IgA Nephropathy Network, Chinese Preventive Medicine Association’s Committee for the Prevention and Control of Kidney Diseases, 2025; Tsuji et al., 2025). This molecular context provides a more specific biological substrate for the “heat manifestations and inflammatory activation” and “heat forcing blood circulation and microenvironment stress” of Blood-Heat syndrome, allowing syndrome research to be grounded at the level of measurable immune-inflammatory networks. Furthermore, at the research design level, the comorbidity spectrum in pediatric cohorts is generally more limited than in adults, which helps to reduce confounding from long-term chronic disease exposures and allows for a more focused investigation of the biological differences inherent to the “syndrome” itself.

The significance of this study lies in providing quantifiable, objective evidence for the precise syndrome differentiation of HSPN by systematically analyzing the biological characteristics of Blood-Heat syndrome and, using HSPN as a paradigm, exploring the common biological basis of Blood-Heat syndrome. Based on this, we integrated clinical serum proteomics analysis with validation in a disease-syndrome combination animal model. We aimed to answer the core question, “What are the objective molecular characteristics of Blood-Heat syndrome?” from a molecular perspective, to screen and validate biomarkers for syndrome identification, and thereby to promote the transformation of the syndrome concept into a measurable pathophysiological state.

## 2 Materials and methods

### 2.1 Clinical study

This study included a discovery cohort and a validation cohort. All research was approved by the Ethics Committee of the First Affiliated Hospital of Henan University of Chinese Medicine (Approval No. 2024HL-187-01). Informed consent was obtained from all subjects or their legal guardians.

For diagnostic criteria, Western medicine diagnosis referred to The Subspecialty Group of Nephrology, the Society of Pediatrics, Chinese Medical Association (2017). The diagnosis of HSPN was made if hematuria and/or proteinuria occurred within 6 months of the onset of Henoch-Schönlein Purpura (HSP). Hematuria was defined by either: (1) gross hematuria; or (2) microscopic hematuria with ≥3 red blood cells per high-power field (HPF) on three occasions within 1 week. Proteinuria was defined by either: (1) positive urine protein on three routine urinalyses within 1 week; (2) 24-h urinary protein excretion >150 mg; (3) urine protein/creatinine ratio >0.2 (mg/mg); or (4) urinary microalbumin higher than normal on three occasions within 1 week.

TCM syndrome differentiation was based on the Guidelines for Ding et al. (2011) and the syndrome differentiation criteria of National Master of Chinese Medicine, Professor Ding Ying. The diagnosis of Blood-Heat syndrome required a combination of major and minor symptoms. Major symptoms included at least two of the following: (1) abundant, bright red skin purpura; (2) vexation; (3) dry mouth with a desire to drink; (4) hematochezia or hematuria. Minor symptoms included at least one of the following, with tongue and pulse findings being mandatory: (1) flushed face and red lips; (2) constipation; (3) red or crimson tongue; (4) thin yellow or thick yellow tongue coating; (5) rapid and forceful pulse.

Regarding study subjects, the discovery cohort enrolled 45 children with HSPN from April to October 2024, who were divided into the HSPN Blood-Heat syndrome group (HSPNXR, n = 15) and the HSPN non-Blood-Heat syndrome group (HSPNFR, n = 30). Healthy children undergoing physical examinations were included as the control group (ZC, n = 30). The validation cohort, enrolled from October 2024 to January 2025, included an HSPNXR group (n = 30) and an HSPNFR group (n = 30), with no overlap with the discovery cohort, while the control group was shared.

Inclusion criteria for patients were: (1) meeting the diagnostic criteria for HSPN, classified as hematuria and proteinuria type; (2) meeting the TCM diagnostic criteria for Blood-Heat syndrome; (3) age 4–16 years; (4) first onset with a disease course within 6 months, and no standardized treatment with immunosuppressants such as Tripterygium wilfordii polyglycoside tablets, glucocorticoids, or cyclophosphamide; (5) complete clinical history data; (6) informed consent signed by the patient or legal guardian. Patients meeting criteria (1), (2), (3), (4), (5), and (6) were included in the HSPNXR group, while those meeting (1), (3), (4), (5), and (6) were included in the HSPNFR group. Inclusion criteria for healthy volunteers were: (1) generally healthy with no substantive lesions in major organ systems (heart, brain, kidney, lung, etc.), no relevant family history, and normal blood count, liver/kidney function, and urinalysis; (2) age 4–16 years; (3) no history of infection or medication in the past week; (4) informed consent signed by the volunteer or legal guardian.

Exclusion criteria included: (1) age outside the specified range; (2) presence of severe systemic diseases (cardiac, cerebral, hepatic, etc.) or psychiatric disorders; (3) presence of conditions that could cause abnormal urinalysis, such as urinary tract infection, urolithiasis, or hypercalciuria; (4) presence of other kidney diseases such as primary IgA nephropathy, lupus nephritis, hepatitis B virus-associated nephritis, Alport syndrome, or urinary tract tumors, or other conditions such as immune thrombocytopenic purpura or ANCA-associated vasculitis; (5) blood samples that were contaminated, damaged, or failed quality control during collection, processing, or storage.

For proteomic analysis, fasting morning serum samples were collected from all subjects. Serum samples from the discovery cohort were analyzed using data-independent acquisition (DIA) proteomics. The workflow included enrichment of low-abundance proteins with magnetic beads, followed by reduction, alkylation, and trypsin digestion. Data were acquired using nano LC-MS/MS (Vanquish neo and Astral), and data retrieval, qualitative, and quantitative analysis were performed using Spectronaut software. The criteria for identifying differentially expressed proteins (DEPs) were set at a fold change ≥1.2 or ≤0.83 and a *P*-value <0.05.

For biomarker validation, the serum concentrations of candidate biomarkers (AHSG, HRG, KNG1, HP, AZGP1, PTX3, MAPK1, A1BG, COL1A1) were quantified in both the discovery cohort and the validation cohort using enzyme-linked immunosorbent assay (ELISA). The kits were from Cloud-Clone Corp., and procedures were performed strictly according to the manufacturer’s instructions.

### 2.2 Animal experiments

Specific-Pathogen-Free (SPF) grade Wistar male juvenile rats (n = 62, 4 weeks, 80 g–130 g) were purchased from Charles River [License No. SCXK (Zhejiang) 2020-0002]. The animals were housed (18 °C–24 °C temperature, 40%–60% humidity) in the Laboratory Animal Center of Henan University of Chinese Medicine under SPF conditions at a 12 h light/dark cycle, with free drinking water and food provided. All mice were adaptively fed for 1 week before the experiment. This study was approved by the Ethics Committee of Henan University of Chinese Medicine (Approval No. IACUC-202408030).

The modeling approach was adapted and optimized from protocols previously established by our team (Xu et al., 2022). The model group (n = 50) was administered a decoction of heat-natured Chinese herbs (dried ginger, pepper, and long pepper) by gavage daily for 4 weeks to establish an internal environment of Blood-Heat. Starting from the second week, an emulsion of ovalbumin (OVA) and Freund’s complete adjuvant was injected intraperitoneally once a week for 3 weeks. After the final intraperitoneal injection, an allergic reaction was induced by multiple tail vein and subcutaneous injections of OVA solution. The control group (n = 12) received an equivalent volume of purified water.

Model evaluation was conducted using a multidimensional index system. (1) Behavioral system: observation of mental state, color and morphology of skin rashes, color of ears, nose, tongue, and paws, body temperature, water intake, and urination/defecation. (2) Biochemical index system: measurement of 24-h urinary protein and red blood cell count, serum and renal tissue levels of inflammatory factors IL-6 and TNF-α, and activity of energy metabolism-related enzymes, including Na+-K+-ATPase, Ca2+-Mg2+-ATPase, lactate dehydrogenase (LDH), and succinate dehydrogenase (SDH). (3) Pathological index system: morphological changes in skin and kidney tissues were observed using HE and PAS staining; IgA deposition in skin and kidney was detected by immunofluorescence. (4) Molecular biomarker validation: expression levels of the nine candidate biomarkers in the serum and renal tissue of model rats were measured by ELISA.

### 2.3 Statistical analysis

Data processing and graphing were performed using SPSS 26.0 and GraphPad Prism 8.0.2. Categorical data were analyzed using the chi-square test. Depending on the results of normality and homogeneity of variance tests, quantitative data were analyzed using the independent samples t-test, one-way analysis of variance (ANOVA), or non-parametric tests (Mann-Whitney U test or Kruskal–Wallis test). GO and KEGG functional enrichment analyses were performed using the DAVID tool. PPI network analysis was conducted using the STRING database and Cytoscape software. ROC analysis was performed using R packages to evaluate diagnostic efficacy. All tests were two-sided, and a *P*-value <0.05 was considered statistically significant.

## 3 Results

### 3.1 Baseline characteristics of clinical subjects

In both the discovery and validation cohorts, there were no statistically significant differences in the distribution of sex, age, weight, or height when comparing the HSPN Blood-Heat syndrome group (HSPNXR) with the healthy control group (ZC) and the HSPN non-Blood-Heat syndrome group (HSPNFR) (*P* > 0.05). There were also no significant differences in sex, age, weight, height, or disease course between the HSPNXR and HSPNFR groups (*P* > 0.05) (Tables 1–6).

**TABLE 1:** Comparison of baseline characteristics between the ZC group and the HSPNXR group in the discovery cohort.

Variable	ZC group (n = 30)	HSPNXR group (n = 15)	*P*	Statistic
Age (years)	8.87 ± 1.78	8.93 ± 2.28	0.915	−0.108
Gender				
Boys	20 (66.6%)	6 (40%)	0.088	2.915
Girls	10 (33.3%)	9 (60%)		
Body Weight (kg)	34.17 ± 8.57	33.46 ± 8.52	0.796	0.26
Height (cm)	141.97 ± 13.9	141.00 ± 11.18	0.816	0.234

**TABLE 2:** Comparison of baseline characteristics between the ZC group and the HSPNFR group in the discovery cohort.

Variable	ZC group (n = 30)	HSPNFR group (n = 30)	*P*	Statistic
Age (years)	8.87 ± 1.78	8.97 ± 3.33	0.885	−0.145
Gender				
Boys	20 (66.6%)	16 (53.3%)	0.292	1.111
Girls	10 (33.3%)	14 (46.7%)		
Body Weight (kg)	34.17 ± 8.57	30.38 ± 10.57	0.133	1.524
Height (cm)	141.97 ± 3.9	135.70 ± 19.63	0.159	1.427

**TABLE 3:** Comparison of baseline characteristics between the HSPNXR group and the HSPNFR group in the discovery cohort.

Variable	HSPNXR group (n = 15)	HSPNFR group (n = 30)	*P*	Statistic
Age (years)	8.93 ± 2.28	8.97 ± 3.33	0.972	−0.035
Gender				
Boys	6 (40%)	16 (53.3%)	0.399	0.711
Girls	9 (60%)	14 (46.7%)		
Body Weight (kg)	141.00 ± 11.18	135.70 ± 19.63	0.256	1.152
Height (cm)	33.46 ± 8.52	30.38 ± 10.57	0.333	0.979
Variable	15 (11,45)	30 (20,47.25)	0.196	−1.293

**TABLE 4:** Comparison of baseline characteristics between the ZC group and the HSPNXR group in the validation cohort.

Variable	ZC Group (n = 30)	HSPNXR group (n = 30)	*P*	Statistic
Age (years)	8.87 ± 1.78	9.40 ± 2.33	0.323	−0.998
Gender				
Boys	20 (66.6%)	18 (60%)	0.592	0.287
Girls	10 (33.3%)	12 (40%)		
Body Weight (kg)	34.17 ± 8.57	30.70 ± 8.14	0.114	1.606
Height (cm)	141.97 ± 13.9	141.57 ± 14.57	0.914	0.109

**TABLE 5:** Comparison of baseline characteristics between the ZC group and the HSPNFR group in the validation cohort.

Variable	ZC Group (n = 30)	HSPNFR Group (n = 30)	*P*	Statistic
Age (years)	8.87 ± 1.78	10.2 ± 3.32	0.059	−1.942
Gender				
Boys	20 (66.6%)	19 (63.3%)	0.787	0.073
Girls	10 (33.3%)	11 (36.7%)		
Body Weight (kg)	34.17 ± 8.57	34.80 ± 9.44	0.786	−0.273
Height (cm)	141.97 ± 13.90	145.13 ± 20.73	0.49	−0.695

**TABLE 6:** Comparison of baseline characteristics between the HSPNXR group and the HSPNFR group in the validation cohort.

Variable	HSPNXR Group (n = 30)	HSPNFR Group (n = 30)	*P*	Statistic
Age (years)	9.70 ± 2.52	10.93 ± 3.38	0.262	−1.133
Gender				
Boys	18 (60%)	19 (63.3%)	0.791	0.071
Girls	12 (40%)	11 (36.7%)		
Body Weight (kg)	30.70 ± 8.14	35.10 ± 9.43	0.077	−1.801
Height (cm)	141.57 ± 14.57	145.47 ± 20.47	0.444	−0.771
Variable	30 (18,60)	30 (20,90)	0.518	−0.647

### 3.2 Proteomic profile of HSPN patients with Blood-Heat syndrome suggests alterations in multiple inflammatory pathways and enrichment related to sphingolipid signaling

**FIGURE 1:**
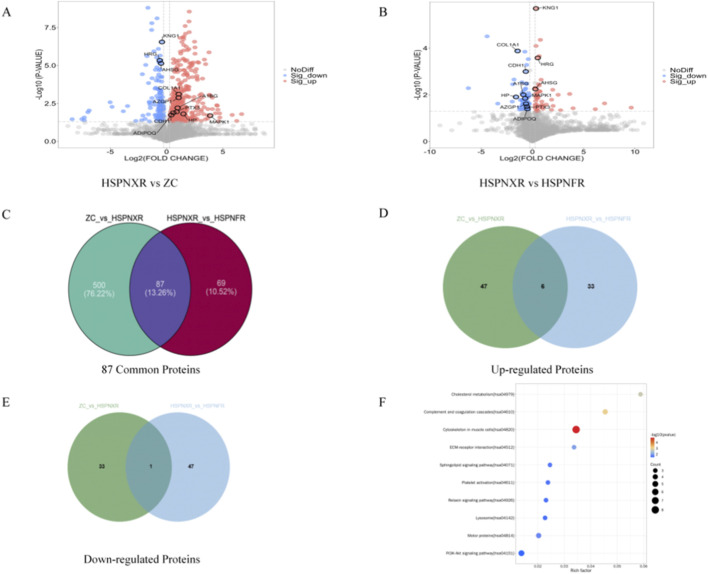
Proteomic profile analysis of HSPN Blood-Heat syndrome. **(A)** Volcano plot comparing the HSPNXR group and the ZC group. **(B)** Volcano plot comparing the HSPNXR group and the HSPNFR group. **(C)** Venn diagram of common DEPs from the two comparisons. **(D)** Venn diagram of upregulated proteins among 87 common DEPs. **(E)** Venn diagram of downregulated proteins among 87 common DEPs. **(F)** Bubble chart of KEGG enriched pathways for the 87 common DEPs. Note: The x-axis represents the log_2_-transformed fold change of each protein between the compared groups, indicating up- or downregulation based on the positive or negative value of Log_2_FC; The y-axis represents the negative logarithm (base 10) of the P-value obtained from statistical testing (t-test or chi-square test), i.e., -log_10_ (P-value); The color of the points indicates the final DEP screening result: significantly upregulated DEPs are marked in red, significantly downregulated DEPs are marked in blue, and proteins that did not reach the significance threshold are marked in gray. The size of the bubble represents the number of enriched proteins, and the color intensity represents the level of significance (*P*-value).

Proteomic analysis of serum samples from the discovery cohort identified a total of 2,216 proteins. To screen for proteins directly related to Blood-Heat syndrome, we performed pairwise comparisons. Compared to the ZC group, a total of 587 DEPs were identified in the HSPNXR group, with 446 upregulated and 141 downregulated. Compared to the HSPNFR group, 156 DEPs were identified in the HSPNXR group, with 75 upregulated and 81 downregulated (Figures 1A,B).

To further focus on the molecular changes associated with the Blood-Heat syndrome phenotype, we took the intersection of the DEPs from the two comparisons, yielding 87 common DEPs (Figure 1C). We also plotted Venn diagrams for upregulated and downregulated proteins, respectively (Figures 1D,E). This result provided a set of candidate differential proteins for subsequent functional annotation, network analysis, and biomarker screening. KEGG pathway enrichment analysis of these 87 common DEPs showed that they were significantly enriched in 23 signaling pathways, including the Sphingolipid signaling pathway (hsa04071, *P* = 0.02), Complement and coagulation cascades (hsa04610, *P* < 0.01), and the PI3K/Akt signaling pathway (hsa04151, *P* = 0.03) (Figure 1F). Based on the nature of the enrichment results, a more cautious statement is that the differential proteins related to Blood-Heat syndrome show a dispersed enrichment in multiple pathways related to inflammation, metabolism, and structural remodeling, including the sphingolipid signaling pathway, suggesting it warrants further investigation in future mechanistic studies.

### 3.3 Screening of core biomarkers for HSPN Blood-Heat syndrome

**FIGURE 2:**
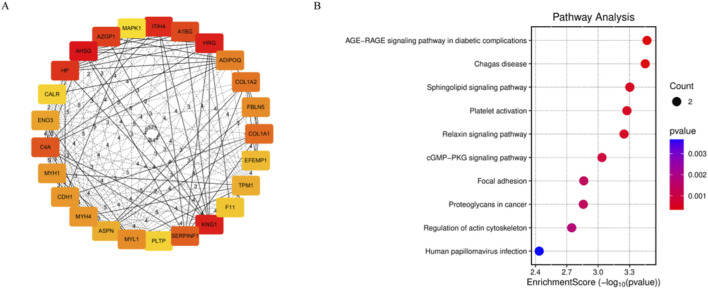
PPI Network analysis and KEGG enrichment pathway bubble plot for the nine biomarkers. **(A)** Protein-protein interaction (PPI) network analysis diagram of the top 25 hub genes. **(B)** KEGG pathway enrichment bubble plot for the nine identified HSPN Blood-Heat Syndrome biomarkers.

To identify the most representative core biomarkers from the 87 candidate proteins, we constructed a protein-protein interaction (PPI) network. Proteins were ranked using the Maximal Clique Centrality (MCC) algorithm based on their network importance, and their diagnostic performance was evaluated by Receiver Operating Characteristic (ROC) curve analysis to obtain the Area Under the Curve (AUC) values. Based on the MCC scores, the top 25 hub proteins occupying critical topological positions within the PPI network were screened (Figure 2A), including proteins such as AZGP1 and HP. Among these, AZGP1 and HP have been previously reported to be associated with the pathogenesis of Henoch-Schönlein Purpura Nephritis (HSPN) and were considered potential biomarkers for HSPN (Zhu et al., 2023; Bajželj et al., 2025). Furthermore, among the initial 87 DEPs, PTX3 has also been documented in the literature as a potential HSPN biomarker (Ge et al., 2014).

Consequently, PTX3 was added to the list, resulting in a total of 26 hub proteins for further evaluation. By integrating the MCC scores, AUC values, and supporting literature evidence (Zhu et al., 2023; Bajželj et al., 2025; Ge et al., 2014), we ultimately selected nine DEPs as biomarkers for the Blood-Heat Syndrome of HSPN from the aforementioned 26 proteins: Alpha-2-HS-glycoprotein (AHSG,MCC score: 975), Histidine-rich glycoprotein (HRG, MCC score:968), Kininogen-1 (KNG1, MCC score:968), Haptoglobin (HP, MCC score:853), Zinc-α2-glycoprotein (AZGP1, MCC score:727), Alpha-1B-glycoprotein (A1BG, MCC score:720), and Collagen Type I alpha 1 Chain (COL1A1, MCC score:22), Mitogen-Activated Protein Kinase 1 (MAPK1, MCC score:4), Pentraxin 3 (PTX3). Further KEGG pathway enrichment analysis of these nine biomarkers also revealed significant enrichment in the sphingolipid signaling pathway (Figure 2B).

**FIGURE 3:**
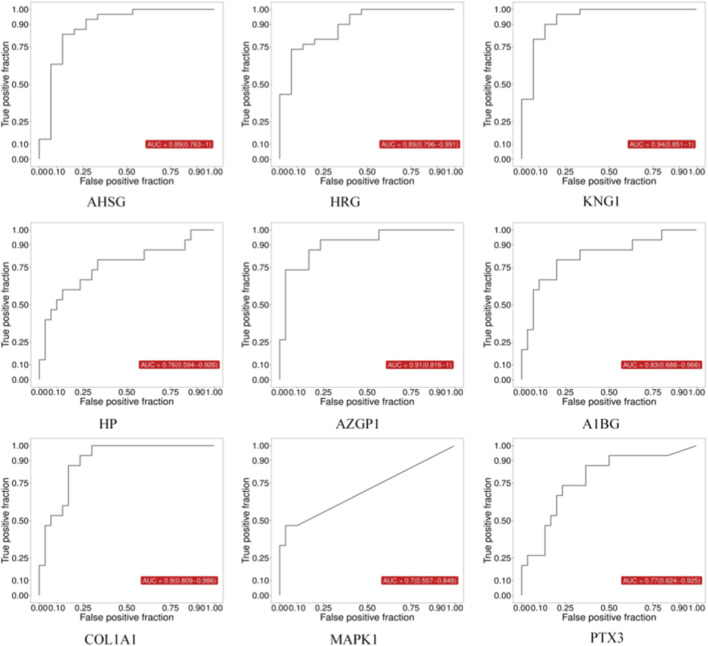
ROC analysis of 9 candidate biomarkers (ZC group vs. HSPNXR group).

**FIGURE 4:**
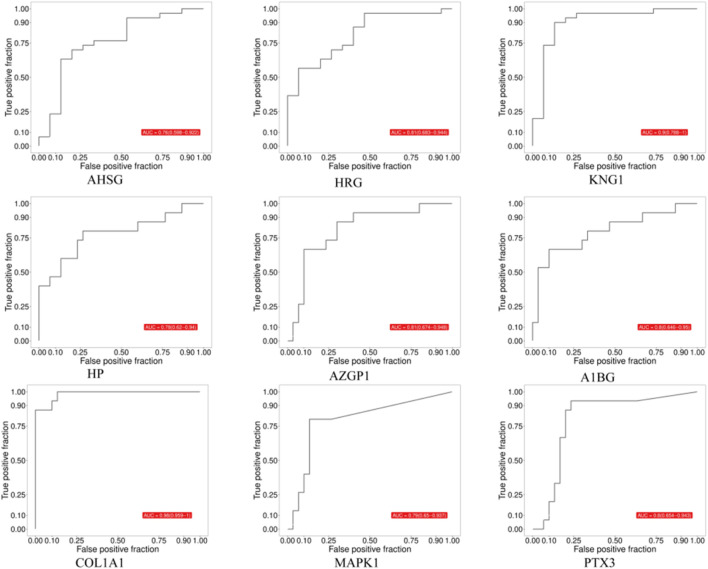
ROC analysis of 9 candidate biomarkers (HSPNXR group vs. HSPNFR group).

**FIGURE 5:**
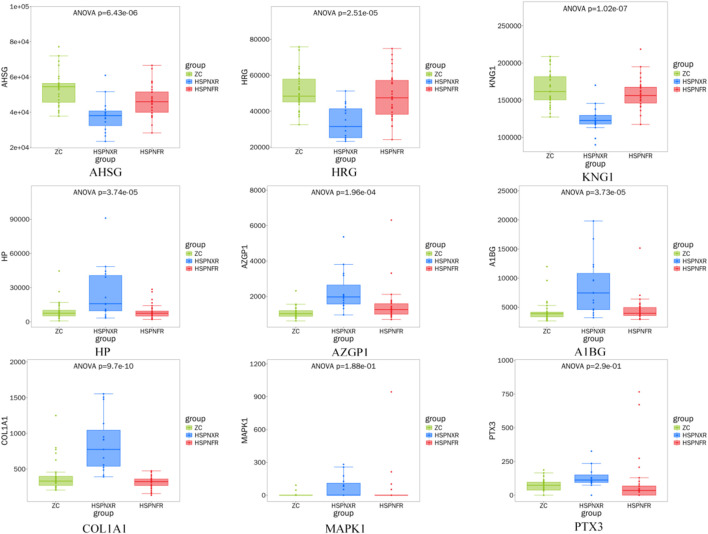
Proteomic expression trends of 9 candidate biomarkers in the discovery cohort. Note: ZC group (n = 30), HSPNXR group (n = 15),HSPNFR group (n = 30).

ROC analysis showed that all nine biomarkers exhibited good diagnostic potential in distinguishing between the normal healthy group and HSPN with Blood-Heat syndrome, as well as HSPN Blood-Heat syndrome from non-Blood-Heat syndrome (all AUC ≥0.7, Figures 3, 4; Table 7). Among them, COL1A1 and KNG1 showed particularly outstanding diagnostic efficacy (all AUC > 0.9). Proteomic data revealed that, compared with the normal control (ZC) group, the expression levels of AHSG, HRG, and KNG1 were downregulated, while those of HP, AZGP1, PTX3, MAPK1, A1BG, and COL1A1 were upregulated in the HSPNXR group. Furthermore, within the HSPNXR group, the expression levels of AHSG, HRG, and KNG1 were significantly lower than those in the HSPNFR group, whereas the remaining six proteins showed significantly higher expression (Figure 5; Table 1).

**TABLE 7:** Diagnostic efficacy and expression trends of the 9 core biomarkers in blood-heat syndrome.

Biomarker	Full name	Expression trend in blood-heat syndrome	AUC (95% CI) (ZC vs. HSPNXR)	AUC (95% CI) (HSPNXR vs. HSPNFR)
AHSG	Alpha-2-HS-glycoprotein	↓ Downregulated	0.89 (0.763–1)	0.76 (0.598–0.922)
HRG	Histidine-rich glycoprotein	↓ Downregulated	0.89 (0.796–0.991)	0.81 (0.683–0.944)
KNG1	Kininogen-1	↓ Downregulated	0.94 (0.851–1)	0.90 (0.788–1.000)
HP	Haptoglobin	↑ Upregulated	0.76 (0.594–0.926)	0.78 (0.620–0.940)
AZGP1	Zinc-alpha-2-glycoprotein	↑ Upregulated	0.91 (0.816–1)	0.81 (0.674–0.948)
A1BG	Alpha-1B-glycoprotein	↑ Upregulated	0.83 (0.688–0.966)	0.80 (0.646–0.950)
COL1A1	Collagen type I alpha 1 chain	↑ Upregulated	0.9 (0.809–0.986)	0.98 (0.959–1.000)
MAPK1	Mitogen-activated protein kinase 1	↑ Upregulated	0.7 (0.557–0.848)	0.79 (0.650–0.937)
PTX3	Pentraxin 3	↑ Upregulated	0.77 (0.624–0.925)	0.80 (0.654–0.943)

### 3.4 Clinical validation of core biomarkers for HSPN Blood-Heat syndrome

**FIGURE 6:**
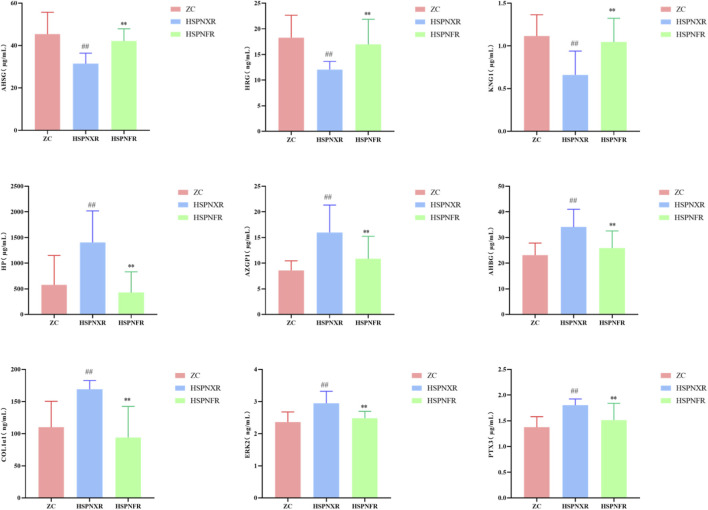
ELISA detection levels of the 9 core biomarkers in the discovery cohort. Note: Comparison between HSPNXR group and ZC group, ^##^ *P* < 0.01; Comparison between HSPNXR group and HSPNFR group, *P < 0.05, **P < 0.01. ZC group (n = 30), HSPNXR group (n = 15), HSPNFR group (n = 30).

**FIGURE 7:**
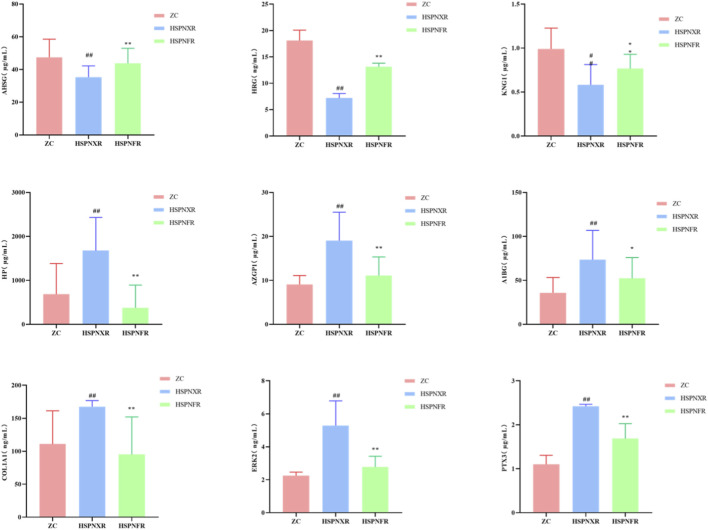
ELISA detection levels of the 9 core biomarkers in the validation cohort. Note: Comparison between HSPNXR group and ZC group, ^##^ *P* < 0.01; Comparison between HSPNXR group and HSPNFR group, ^*^ *P* < 0.05, ^**^ *P* < 0.01. ZC group (n = 30), HSPNXR group (n = 30), HSPNFR group (n = 30).

To validate the above findings, we measured the serum concentrations of these nine markers using ELISA method in both the discovery cohort and an independent clinical validation cohort. The results were highly consistent with the proteomic data: Compared to the ZC and HSPNFR groups, the concentrations of AHSG, HRG, and KNG1 in the HSPNXR group were significantly decreased, while the concentrations of HP, AZGP1, A1BG, COL1A1, MAPK1 (also known as ERK2), and PTX3 were all significantly increased (*P* < 0.01 for all comparisons) (Figures 6, 7). To further validate the diagnostic value of the identified core biomarkers, we performed a combined ROC analysis using ELISA data from both the discovery and validation cohorts. The results showed that the AUC values of these nine biomarkers were all greater than 0.74 between the ZC group and the HSPNXR group, and greater than 0.82 between the HSPNXR group and the HSPNFR group (Supplementary Material 2). This result strongly confirms the reliability of these nine proteins as biomarkers for HSPN Blood-Heat syndrome.

### 3.5 The HSPN Blood-Heat syndrome animal model successfully replicated clinical biological features

**FIGURE 8:**
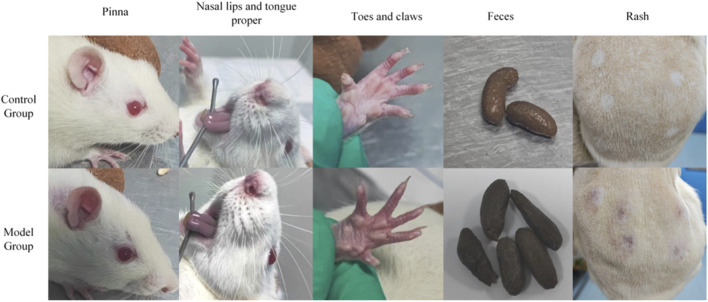
Phenotypic signs of rats in the control and model groups after modeling.

**FIGURE 9:**
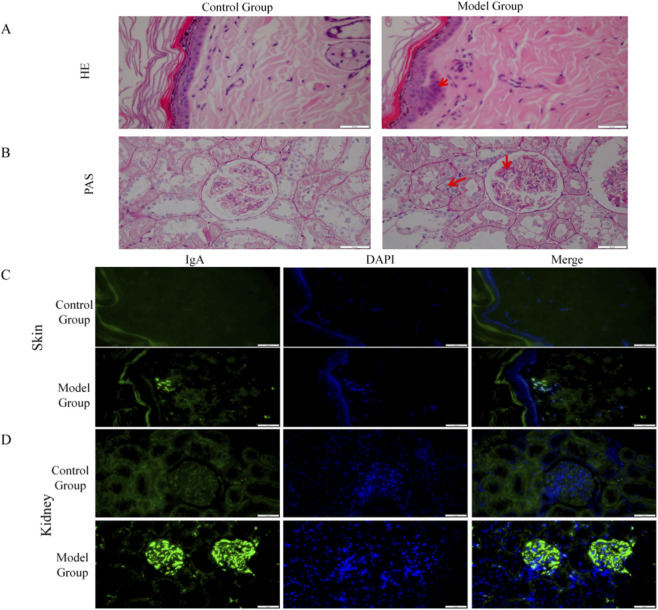
Pathological conditions of skin and kidney tissues in the control and model groups after modeling. **(A)** HE staining of skin tissues in the Control and Model groups after Modeling. **(B)** PAS staining of kidney tissues in the Control and Model groups after Modeling. **(C)** IgA Immunofluorescence Deposition in Skin Tissues of the Control and Model groups after Modeling. **(D)** IgA immunofluorescence deposition in kidney tissues of the Control and Model groups after modeling. Note: Inflammatory cell infiltration and mesangial proliferation are indicated by arrows.

To validate the biological characteristics of Blood-Heat syndrome at the *in vivo* level, we successfully established a rat model combining HSPN disease with Blood-Heat syndrome. Compared to the control group, rats in the model group exhibited red auricles, nose, lips, and tongue, were irritable and aggressive, had scanty and yellow urine, and dry stools, accompanied by decreased body weight, increased rectal temperature, and increased 24-h water intake (Supplementary Material 3). Body weights were 487.43 ± 2.78 g vs. 509.17 ± 26.82 g (*P* < 0.05), rectal temperatures were 38.77 °C ± 0.39 °C vs. 37.92 °C ± 0.54 °C (*P* < 0.01), and 24-h water intake was 56.22 ± 12.48 mL vs. 42.00 ± 8.67 mL (*P* < 0.01), respectively (Figure 8, Supplementary Material 3). Concurrently, the model rats also displayed typical clinical manifestations and pathological features of HSPN, including skin purpura, significantly elevated 24-h urinary protein and red blood cell counts (*P* < 0.01) (Table 8), as well as inflammatory cell infiltration around small blood vessels in the skin, mesangial proliferation, and IgA immune complex deposition in renal tissue (Figures 9A–D).

**TABLE 8:** Comparison of 24-hour urinary protein quantification and urinary red blood cell counts between the control group and model group before and after Modeling ( $\bar{x}$ ±s).

Group	n	24-Hour urinary protein (mg/24 h)		Urinary RBC Count (/μL)	
		Pre-modeling	Post-modeling	Pre-modeling	Post-modeling
Control group	12	2.32 ± 0.87	13.49 ± 1.67	15.78 ± 2.63	15.58 ± 3.46
Model group	45	2.61 ± 0.81	28.92 ± 5.46^##^	16.03 ± 2.95	37.60 ± 8.94^##^

Compared with the Control group.

^##^ *P* < 0.01.

At the molecular level, the biochemical indicators of the model rats further confirmed their “heat syndrome” state. Compared to the control group, the levels of pro-inflammatory factors IL-6 and TNF-α in the serum and renal tissue of the model group were significantly elevated (*P* < 0.01) (Table 9) (Supplementary Material). Additionally, the activities of energy metabolism-related enzymes reflecting hypermetabolism, including Na+-K+-ATPase, Ca2+-Mg2+-ATPase, LDH, and SDH, were all significantly increased (*P* < 0.05) (Table 10).

**TABLE 9:** Comparison of IL-6 and TNF-α levels in serum and kidney tissues between the control group and model group after modeling ( $\bar{x}$ ±s, n = 3).

Group	IL-6 (ng/mL)		TNF-α(pg/mL)	
	Serum	Kidney	Serum	Kidney
Control group	1.08 ± 0.12	3.11 ± 0.30	126.24 ± 19.43	158.87 ± 12.21
Model group	6.64 ± 1.17^##^	8.12 ± 0.96^##^	412.62 ± 32.12^##^	726.85 ± 116.91^##^

Compared with the Control group.

^##^ *P* < 0.01.

**TABLE 10:** Comparison of energy metabolism enzymes in serum and kidney tissues between the control group and model group after modeling ( $\bar{x}$ ±s, n = 3).

Indicators		Control group	Model group
Na^+^/K^+^-ATPase	Serum	153.65 ± 11.80	179.73 ± 9.03^#^
	Kidney	157.90 ± 7.87	185.59 ± 12.22^#^
Ca^2+^/Mg^2+^-ATPase	Serum	61.37 ± 4.82	74.76 ± 5.54^#^
	Kidney	85.23 ± 3.78	131.44 ± 12.61^##^
LDH activity	Serum	630.66 ± 16.72	690.60 ± 11.97^##^
	Kidney	12.76 ± 1.88	31.31 ± 5.23^##^
SDH activity	Serum	14.50 ± 0.88	21.89 ± 1.91^##^
	Kidney	3.34 ± 0.46	6.27 ± 1.18^#^

Compared with the Control group.

^#^ *P* < 0.05, ^##^ *P* < 0.01.

**FIGURE 10:**
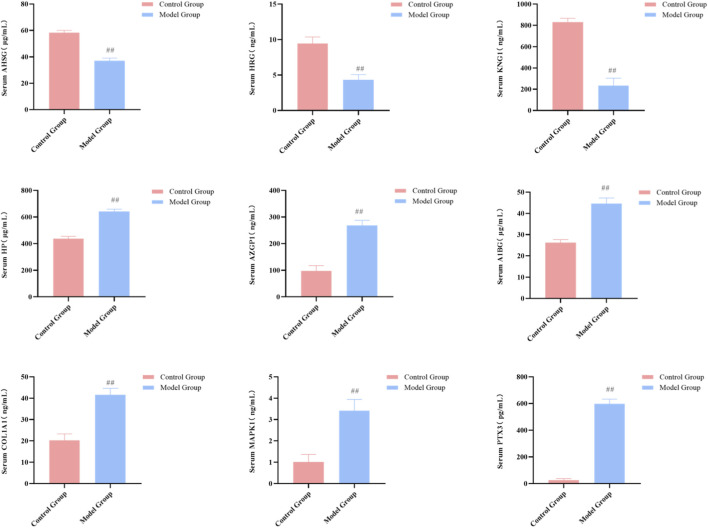
Replication of core biomarkers in the HSPN Blood-Heat syndrome animal model (Serum, n = 7). Note: Comparison between the control group and model group, ^##^ *P* < 0.01.

**FIGURE 11:**
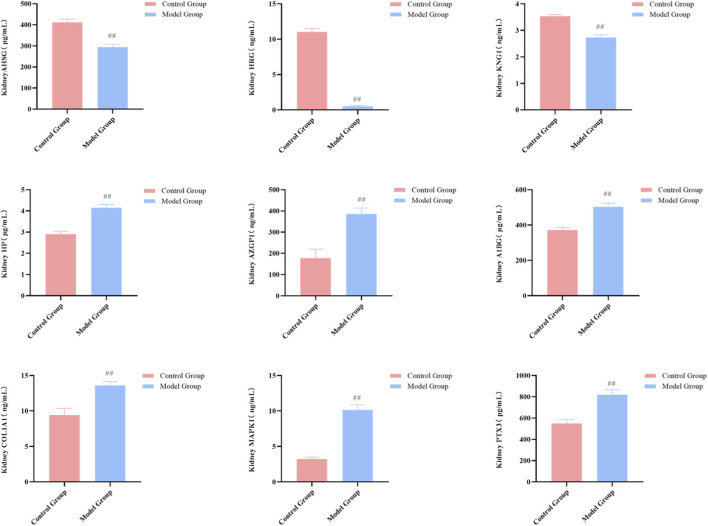
Replication of core biomarkers in the HSPN Blood-Heat syndrome animal model (Kidney Tissue, n = 7). Note: Comparison between the control group and model group, ^##^ *P* < 0.01.

Most critically, the animal model precisely replicated the findings from clinical patients at the molecular biomarker level. ELISA results showed that, compared to the control group, the expression changes of the nine core biomarkers in the serum and renal tissue of the model group were completely consistent with those in HSPN patients with Blood-Heat syndrome: concentrations of AHSG, HRG, and KNG1 were significantly decreased (*P* < 0.01), while concentrations of HP, AZGP1, A1BG, COL1A1, MAPK1 (ERK2), and PTX3 were all significantly increased (*P* < 0.01) (Figures 10, 11). This result provides solid *in vivo* biological validation for the biomarkers discovered clinically, completing the evidence chain from clinical observation to animal model replication.

## 4 Discussion

### 4.1 Multidimensional evidence suggests network rearrangement in inflammation, metabolism, and structural remodeling, with enrichment related to sphingolipid signaling in Blood-Heat syndrome

This study systematically investigated the modern biological basis of the TCM “Blood-Heat syndrome” from multiple dimensions, based on clinical proteomics and a disease-syndrome combination animal model. Clinical observations indicate that there are gender differences in HSPN, with a higher incidence rate in males than in females (Shen et al., 2025). Furthermore, estrogen exerts an anti-inflammatory effect by antagonizing the release of inflammatory mediators, thereby providing multi-organ protection (El Sabeh et al., 2021). Therefore, the use of male animals in this study is considered more suitable for establishing a robust HSPN model.

Our results indicate that HSPN patients with Blood-Heat syndrome exhibit a reproducible serum protein expression profile. The differential proteins are functionally enriched in networks related to inflammation, metabolism, and structural remodeling, particularly involving three key signaling pathways: (1) Sphingolipid signaling pathway—Sphingolipid metabolism and its associated signaling pathways play crucial regulatory roles in various inflammatory diseases and kidney disorders (Li and Li, 2025). Drawing from our team’s prior research (Bai, 2017; Li et al., 2023), non-targeted metabolomic analyses of serum samples from children with HSP Blood-Heat syndrome, as well as from a rat model of HSP with Blood-Heat syndrome, have consistently demonstrated significant enrichment of differentially expressed metabolites in the sphingolipid metabolism pathway. Furthermore, disease progression correlates with alterations in sphingolipid metabolism. Research suggests that disruptions in energy metabolism, primarily involving amino acids, carbohydrates, and lipids, may represent one of the key pathological mechanisms underlying Blood-Heat syndrome (Sun et al., 2025). Based on above, it is reasonable to hypothesize that sphingolipid metabolism and its signaling pathways play a critical role in the molecular mechanisms of kidney injury in HSPN associated with Blood-Heat syndrome. (2) Complement and coagulation cascades—The onset and progression of HSPN are closely linked to complement system activation and coagulation dysfunction. Complement activation can lead to fibrin deposition, glomerular basement membrane disruption, inflammatory cell infiltration, and endothelial cell activation, thereby exacerbating renal tissue damage (Davin and Coppo, 2014). Simultaneously, complement activation and the coagulation cascade form a positive feedback loop (Damman et al., 2022), creating a vicious cycle of inflammation and coagulation. Several recent proteomics studies (e.g., by Fang et al. (2021), Demir et al. (2023)) have validated the enrichment of this pathway in urine and plasma samples, which is highly consistent with our findings and further confirms the pivotal role of the complement-coagulation cascade in HSPN pathogenesis. (3) PI3K/Akt signaling pathway—As a central regulator of cell proliferation, apoptosis, and inflammatory responses, it could modulate autophagy in renal tissue cells, podocyte injury, and inflammation, and is closely implicated in the progression of various kidney diseases (Lu et al., 2020; Dong et al., 2024). The interplay of these pathways may constitute the molecular basis underlying the “extravasation of blood due to blood heat” pathogenesis, in which sphingolipid metabolism and its associated signaling pathways are likely to play a pivotal role.

We also screened and validated a candidate biomarker panel consisting of nine core proteins (AHSG, HRG, KNG1, HP, AZGP1, PTX3, MAPK1, A1BG, COL1A1). Previous research has shown that the omics features of HSPN are highly correlated with prognosis, and integrated analysis of proteomics and multi-omics from body fluids like blood and urine has been used to explore non-invasive biomarkers and key pathway networks (Zhou et al., 2025; Fang et al., 2020; Liu et al., 2023; Gao et al., 2020; Xie et al., 2020).

Against this research background, our study, using “syndrome” as the unit of analysis, performed consistent validation of candidate biomarkers in clinical cohorts and an animal model, thereby providing quantifiable molecular evidence for the objective characterization of Blood-Heat syndrome.

### 4.2 The molecular fingerprint of Blood-Heat syndrome: a network imbalance of downregulated immune homeostasis-related proteins and upregulated stress-related proteins

The nine core biomarkers we identified do not point to a single molecular event but rather present a more systemically significant “molecular fingerprint.” Their common feature can be summarized as a synchronous shift at two ends: one end is the downregulation of proteins related to immune homeostasis and coagulation-inflammation regulation, and the other is the upregulation of proteins related to inflammatory stress, immune activation, and tissue remodeling. This combination is more consistent with an “imbalance in immune-coagulation coupling against a background of immune complex-related inflammation” and provides a testable biological explanatory framework for the objective characterization of Blood-Heat syndrome.

On the downregulated side: (1) AHSG levels can decrease in populations with kidney disease and are inversely correlated with the inflammatory marker hs-CRP, suggesting its changes are related to inflammatory load (Rudloff et al., 2022). Meanwhile, urinary proteomic studies suggest that AHSG-related proteins show characteristic fluctuations with changes in renal function status (Makhammajanov et al., 2024). Therefore, the observed downregulation of AHSG in our study is more likely to indicate a persistent inflammatory stress background in Blood-Heat syndrome, rather than a simple metabolic change (2) HRG can interact with immunoglobulins and various complement system proteins, and can affect immune complex formation and complement-mediated clearance processes (Manderson et al., 2009). Further review evidence indicates that HRG participates in the clearance of immune complexes and apoptotic cells and may affect the persistence of autoimmune-related inflammation (Gorgani and Theofilopoulos, 2007). Given that IgA-related immune complexes carry a complex complement proteome (Tsuji et al., 2025), HRG downregulation signals a reduced capacity to maintain immune complex homeostasis. (3) KNG1 encodes high-molecular-weight kininogen, a key substrate of the contact system bridging intrinsic coagulation and inflammatory amplification via bradykinin release (Emsley et al., 2025). Its downregulation in chronic kidney disease contexts (Palarasah et al., 2025) suggests consumption or remodeling of the coagulation-inflammation coupling axis.

On the upregulated side: (1) HP defends against heme toxicity and endothelial dysfunction by binding free hemoglobin (Macdonald, 2014); its upregulation indicates enhanced oxidative stress and vascular endothelial-related risk. (2) AZGP1 participates in renal lipid metabolism reprogramming and blood pressure regulation (Zhou et al., 2024) and has been included in candidate biomarker panels for kidney disease (Altman et al., 2024). (3) PTX3 is produced by mesangial cells in IgA glomerulonephritis and participates in pro-inflammatory lipid mediator generation (Bussolati et al., 2003; Querin et al., 2025); its upregulation directly reflects an enhanced renal inflammatory microenvironment. (4) MAPK1 (ERK2) is a key kinase node in inflammatory signal transduction that participates in renal inflammatory injury along with NF-κB (Chen et al., 2025); its consistent upregulation across clinical and animal data makes it a priority target for mechanistic validation. (5) A1BG has been reported in urinary proteomic studies as a candidate biomarker related to disease type or renal function (Makhammajanov et al., 2024; Altman et al., 2024; Piyaphanee et al., 2011). (6) COL1A1 is a key molecule related to renal fibrosis and extracellular matrix deposition (Guo et al., 2026); its upregulation suggests a stronger tissue remodeling and fibrosis risk background.

In summary, the “downregulation of AHSG/HRG/KNG1 + upregulation of HP/AZGP1/PTX3/MAPK1(ERK2)/A1BG/COL1A1″ observed in this study is closer to a multi-axis, systemic network imbalance: enhancement of the immune complex and complement-inflammation network, remodeling of the coagulation-inflammation coupling axis, accompanied by a synchronous amplification of metabolic stress and tissue remodeling signals. This explanatory framework is consistent with the modern pathological translation of Blood-Heat syndrome’s “heat manifestations corresponding to inflammatory activation, and heat forcing blood circulation corresponding to microenvironmental stress” (Sun et al., 2025), but the causal hierarchy of each node still needs to be further clarified through intervention and functional experiments.

### 4.3 Enrichment related to sphingolipid signaling and ERK2 upregulation provide testable molecular clues for the inflammatory amplification hypothesis

At the clinical proteomics level, this study observed that the differential proteins related to Blood-Heat syndrome were enriched in the sphingolipid signaling pathway in KEGG analysis, while the key kinase MAPK1 (ERK2) was upregulated in both the clinical cohort and the animal model. It must be emphasized that pathway enrichment essentially reflects the statistical aggregation of candidate differential proteins in existing knowledge base pathway sets and is not equivalent to direct proof of “upstream driving factors.” Therefore, a statement more aligned with the evidence boundary is: the altered protein profile related to Blood-Heat syndrome suggests synergistic fluctuations in multiple inflammation-metabolism-structural remodeling pathways, including the sphingolipid signaling pathway; and the upregulation of ERK2 provides a key node that can be linked to inflammatory signal transduction, helping to construct a testable mechanistic hypothesis.

From the existing research background, sphingolipid metabolism and its key metabolite, sphingosine-1-phosphate (S1P), have been extensively implicated in the regulation of inflammation and kidney diseases (Li and Li, 2025). S1P not only participates in immune cell migration and the shaping of the tissue inflammatory microenvironment but is also associated with multi-organ inflammation and fibrotic processes (Nagahashi et al., 2018; Schwalm et al., 2024). Furthermore, S1P can directly influence multiple pathological features of glomerulonephritis, including mesangial cell proliferation, renal inflammation, and fibrosis (Schwalm et al., 2014; Hait and Maiti, 2017; Li and Li, 2025). The catalytic activity of sphingosine kinase 1 (SPHK1), the rate-limiting enzyme in S1P production, can be directly enhanced by ERK1/2 through phosphorylation (Pitson et al., 2003), thereby tightly coupling upstream inflammatory signals with the sphingolipid metabolic axis. In IgA nephropathy, S1P functions as a gut microbiota-associated metabolite involved in the phenotypic transformation of mesangial cells (Tang et al., 2025) and exerts diverse biological effects through distinct receptor axes (Kurano et al., 2019). Mechanistically, S1P receptors, as members of the G protein-coupled receptor family, can couple with the Akt, p38 MAPK, and ERK signaling pathways (Johansen et al., 2023). Alterations in the SphK/S1P axis are accompanied by upregulation of NF-κB, which can be reversed synchronously upon drug intervention (Crespo et al., 2017). Clinically, S1P receptor modulators have been employed in the treatment of immune-inflammatory diseases to regulate immune cell migration and inflammatory intensity (Shields et al., 2025).

Based on the direct observations of this study and the verifiable evidence above, we are more inclined to define “Blood-Heat-related stress—sphingolipid signaling-associated alterations—ERK2-mediated inflammatory amplification” as a core scientific hypothesis framework, rather than a confirmed pathological axis. Its key verifiable links include: quantifying S1P and related sphingolipid metabolites in future studies, measuring the expression and activity of SPHK1/2 and S1P receptor subtypes, and observing whether inflammatory indicators and tissue damage phenotypes change consistently in animal models through ERK pathway inhibition or S1P signal modulation interventions, thereby advancing the current correlational findings into a more causally explanatory evidence chain.

### 4.4 Systemic advantages and future prospects of the study

This study has several limitations. First, the relatively limited sample size necessitates larger, multi-center validation of the biomarker panel across different pathological types and treatment backgrounds. Second, the use of a lenient fold-change threshold and *P* < 0.05 criterion without systematic multiple comparison correction may introduce false-positive risk. Third, the mechanistic inference is mainly correlation-based; although key node protein changes were observed in the animal model, causal validation requires future intervention strategies such as S1P signal modulation or ERK pathway inhibition. Fourth, the ROC evaluation remains an internal validation; external independent cohort testing following TRIPOD and PROBAST guidelines is needed (Collins et al., 2015; Moons et al., 2025). Fifth, as the evidence is limited to comparing syndromes within HSPN, the current data cannot fully rule out that the observed molecular differences partially reflect a higher overall inflammatory activity rather than syndrome-specific biology; future research should introduce stratification strategies for inflammatory indicators and renal injury degree to further clarify this distinction. Looking ahead, future work could construct a multi-protein-based diagnostic index for stratifying and dynamically monitoring HSPN children, while the sphingolipid signaling-related changes and ERK2 node identified here can serve as priority directions for mechanistic validation and targeted intervention.

## 5 Conclusion

In summary, this study provides new molecular evidence and a systematic perspective on the biological basis of the TCM “Blood-Heat syndrome” in the context of HSPN. Our multidimensional evidence suggests that HSPN Blood-Heat syndrome has a reproducible molecular phenotype, with its proteomic features involving enrichment of the sphingolipid signaling pathway and an enhanced inflammatory background represented by ERK2 upregulation. The nine core proteins identified in this study (AHSG, HRG, KNG1, HP, AZGP1, PTX3, MAPK1, A1BG, COL1A1) collectively constitute a candidate “molecular fingerprint” for this phenotype, providing an operational indicator system and hypothesis framework for the objective characterization and subsequent mechanistic validation of HSPN Blood-Heat syndrome.

## Data Availability

The mass spectrometry proteomics data have been deposited to the ProteomeXchange Consortium (https://proteomecentral.proteomexchange.org) via the iProX partner repository with the dataset identifier PXD076234.
