# Supplementary material for: The biological basis of Blood-Heat syndrome in children with Henoch-Schonlein purpura nephritis: a multidimensional analysis based on clinical proteomics and an animal model
Source: Front Pharmacol. 2026 Apr 10;17:1778919. doi: 10.3389/fphar.2026.1778919 (PMC13105992; doi:10.3389/fphar.2026.1778919)
Supplement: Supplementary file 1 [file Supplementaryfile5.docx]

**Supplementary Table 7. Comparison of 24-Hour Urinary Protein Quantification and Urinary Red Blood Cell Counts Between the Control Group and Model Group Before and After Modeling（**$\bar{\mathbf{x}}$**±s）**

| Group | n | 24-Hour Urinary Protein （mg/24h） | | Urinary RBC Count（/μL） | |
| --- | --- | --- | --- | --- | --- |
|  |  | Pre-modeling | Post-modeling | Pre-modeling | Post-modeling |
| **Control Group** | 12 | 2.32±0.87 | 13.49±1.67 | 15.78±2.63 | 15.58±3.46 |
| **Model Group** | 45 | 2.61±0.81 | 28.92±5.46^##^ | 16.03±2.95 | 37.60±8.94^##^ |

Note: Compared with the **Control** group, ^##^*P*<0.01

**Supplementary Table 8. Comparison of IL-6 and TNF-α Levels in Serum and Kidney Tissues Between the Control Group and Model Group After Modeling（**$\bar{\mathbf{x}}$**±s，n=3）**

| Group | IL-6（ng/ml） | | TNF-α（pg/ml） | |
| --- | --- | --- | --- | --- |
|  | **Serum** | **Kidney** | **Serum** | **Kidney** |
| **Control Group** | 1.08±0.12 | 3.11±0.30 | 126.24±19.43 | 158.87±12.21 |
| **Model Group** | 6.64±1.17^##^ | 8.12±0.96^##^ | 412.62±32.12^##^ | 726.85±116.91^##^ |

Note: Compared with the **Control** group, ^##^*P*<0.01

**Supplementary Table 9. Comparison of Energy Metabolism Enzymes in Serum and Kidney Tissues Between the Control Group and Model Group After Modeling（**$\bar{\mathbf{x}}$**±s，n=3）**

| **Indicators** | | **Control Group** | **Model Group** |
| --- | --- | --- | --- |
| **Na⁺/K⁺-ATPase** | **Serum** | 153.65±11.80 | 179.73±9.03^#^ |
|  | **Kidney** | 157.90±7.87 | 185.59±12.22^#^ |
| **Ca²⁺/Mg²⁺-ATPase** | **Serum** | 61.37±4.82 | 74.76±5.54^#^ |
|  | **Kidney** | 85.23±3.78 | 131.44±12.61^##^ |
| **LDH activity** | **Serum** | 630.66±16.72 | 690.60±11.97^##^ |
|  | **Kidney** | 12.76±1.88 | 31.31±5.23^##^ |
| **SDH activity** | **Serum** | 14.50±0.88 | 21.89±1.91^##^ |
|  | **Kidney** | 3.34±0.46 | 6.27±1.18^#^ |

Note: Compared with the **Control** group, ^##^*P*<0.01
