# Supplementary material for: The biological basis of Blood-Heat syndrome in children with Henoch-Schonlein purpura nephritis: a multidimensional analysis based on clinical proteomics and an animal model
Source: Front Pharmacol. 2026 Apr 10;17:1778919. doi: 10.3389/fphar.2026.1778919 (PMC13105992; doi:10.3389/fphar.2026.1778919)
Supplement: Supplementary file 2 [file Supplementaryfile3.pptx]

## Slide 1
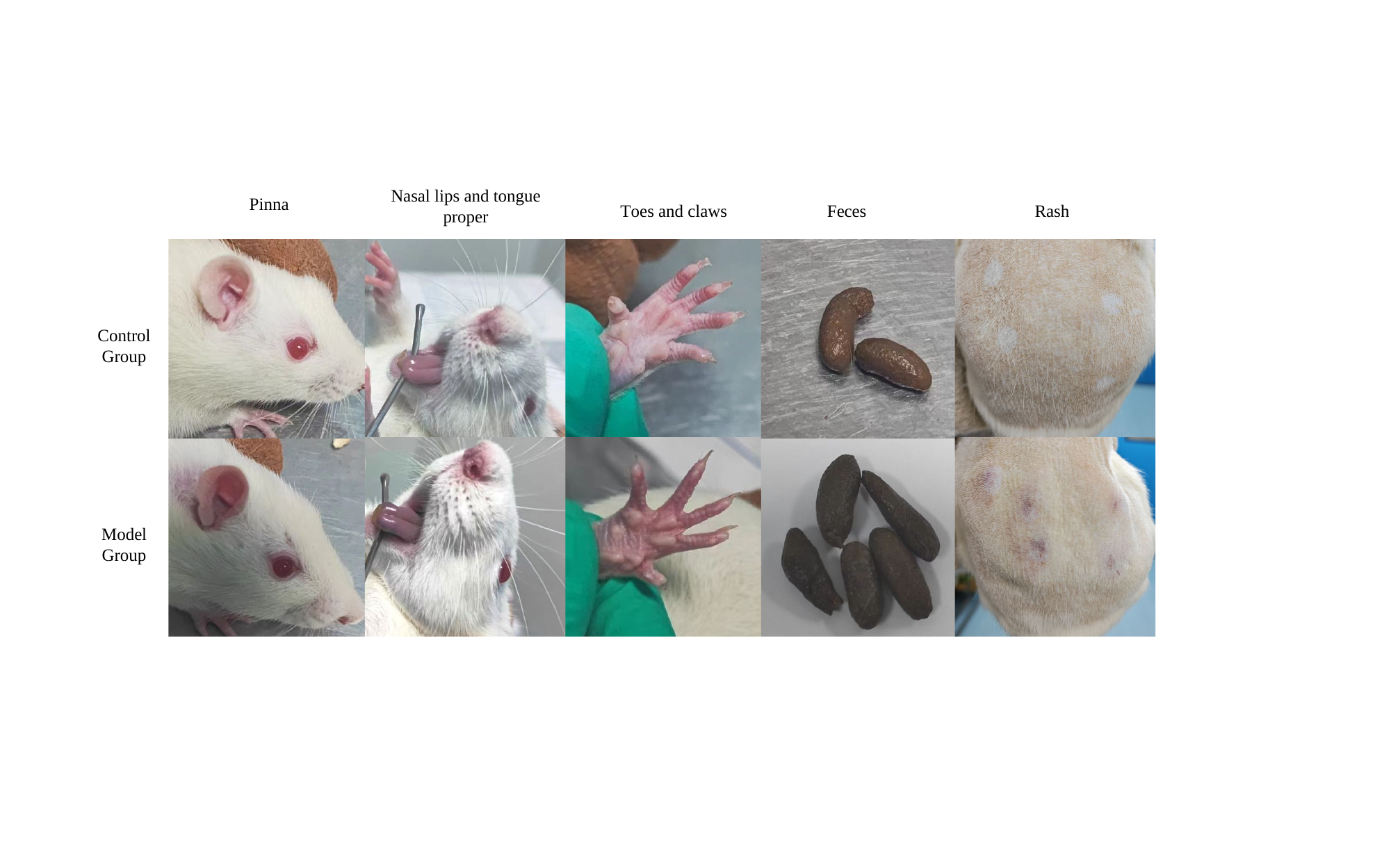

Nasal lips and tongue proper
Pinna
Feces
Rash
Toes and claws
Control
Group
Model Group

## Slide 2
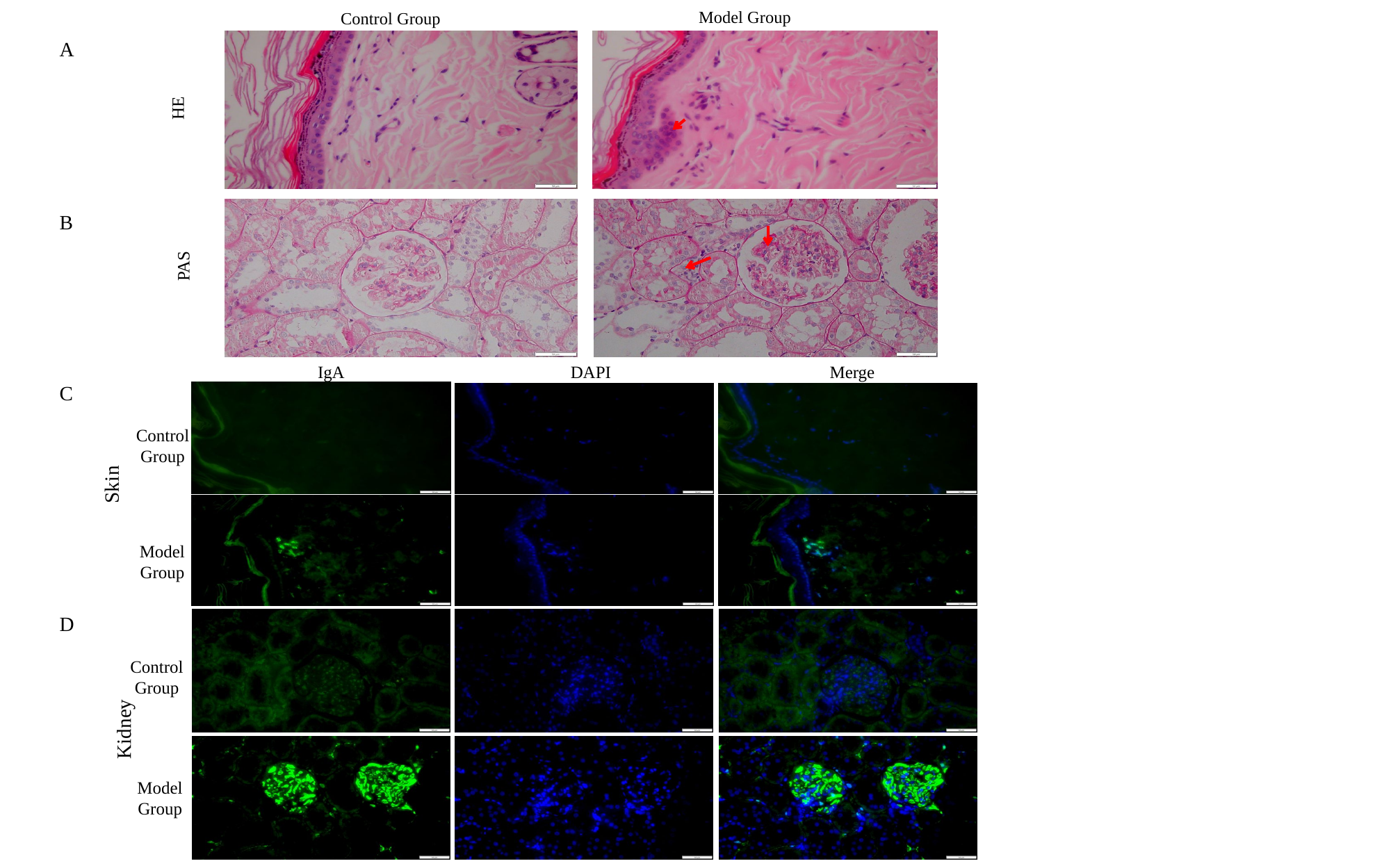

Model Group
Control Group
HE
PAS
A
B
IgA
DAPI
Merge
Control
Group
Model Group
C
 Skin
D
Control
Group
Model Group
 Kidney

## Slide 3
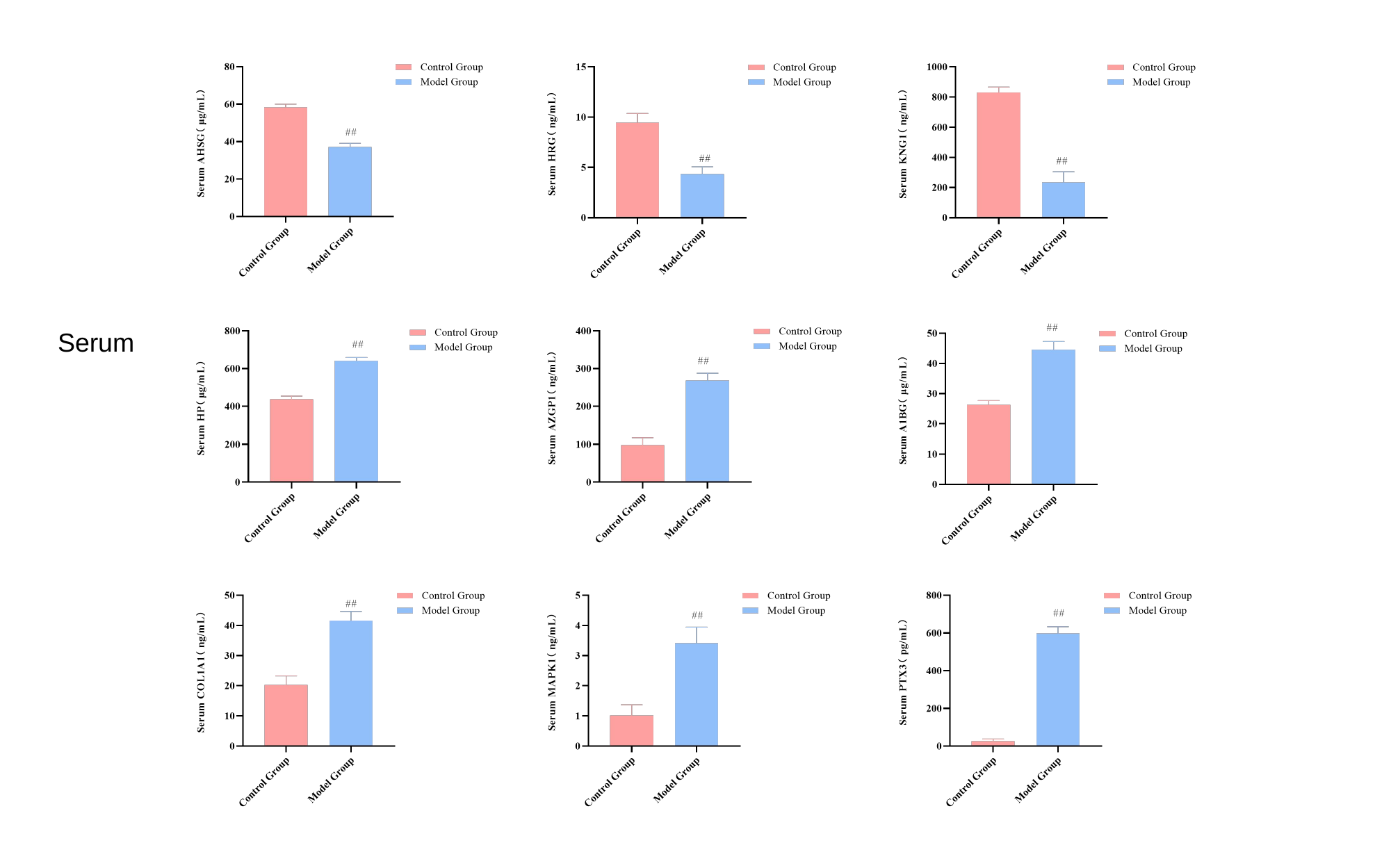

Serum

## Slide 4
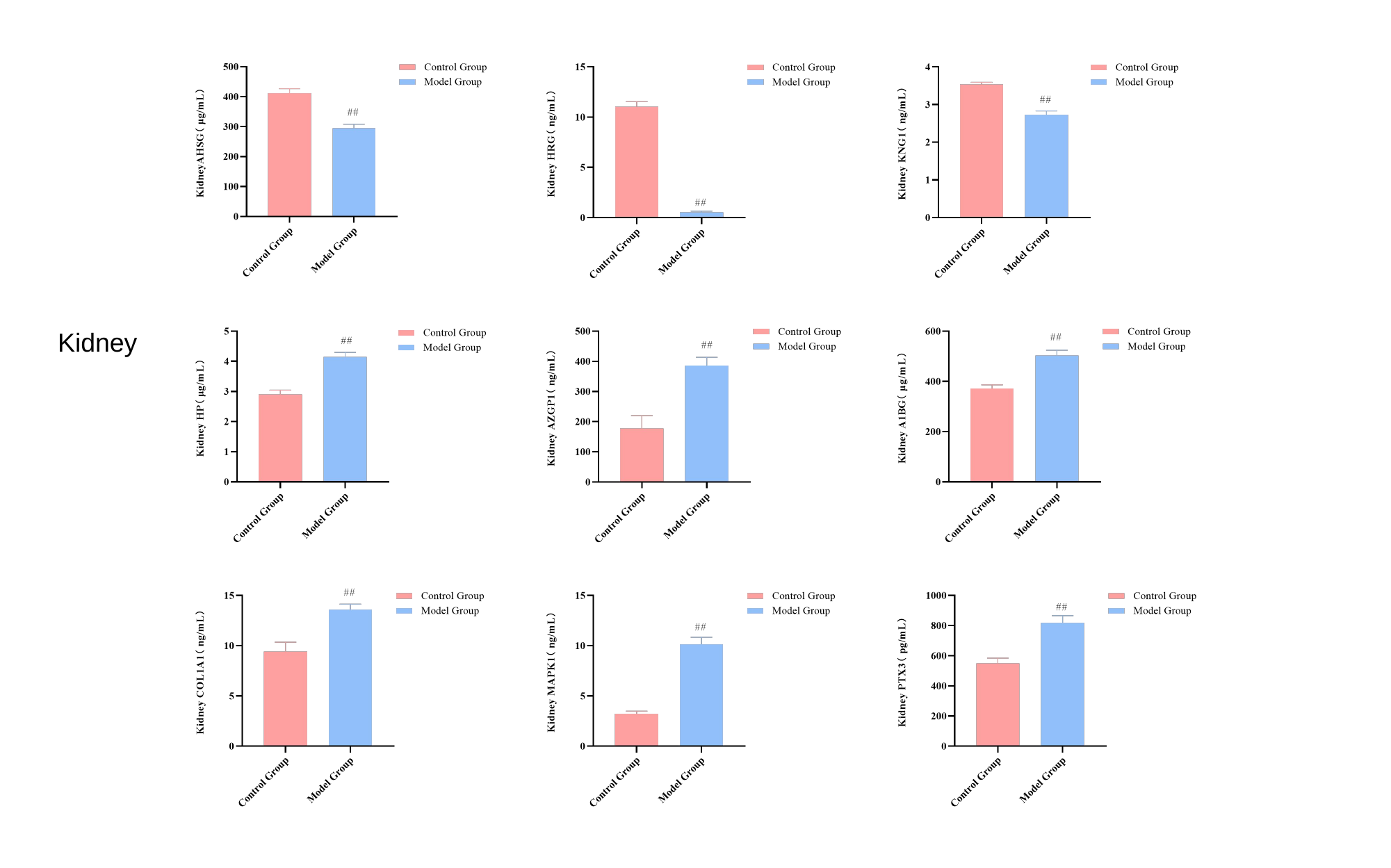

Kidney
