# Supplementary material for: The biological basis of Blood-Heat syndrome in children with Henoch-Schonlein purpura nephritis: a multidimensional analysis based on clinical proteomics and an animal model
Source: Front Pharmacol. 2026 Apr 10;17:1778919. doi: 10.3389/fphar.2026.1778919 (PMC13105992; doi:10.3389/fphar.2026.1778919)

## Slide 1
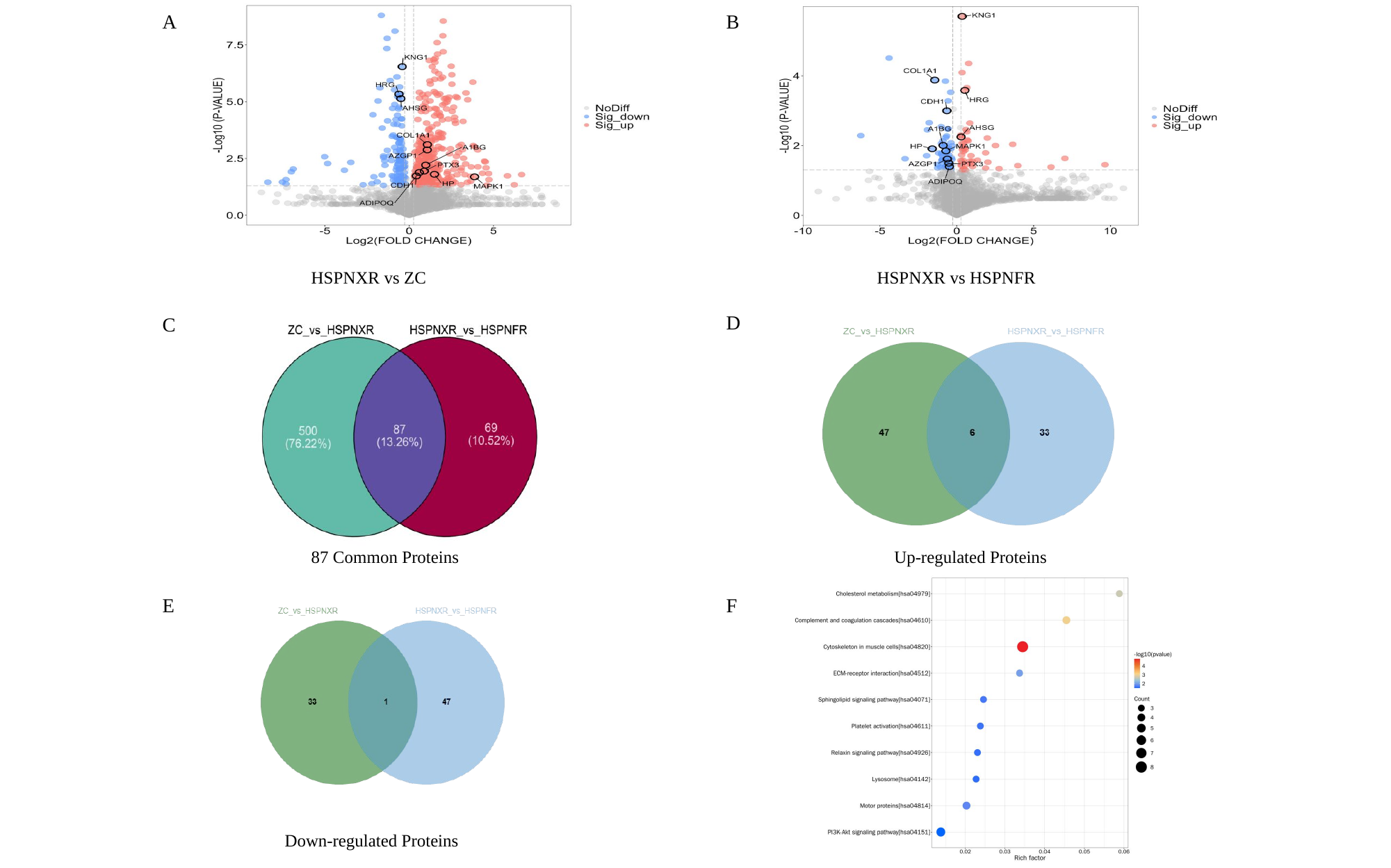

A
B
HSPNXR vs ZC
HSPNXR vs HSPNFR
D
C
87 Common Proteins
Up-regulated Proteins
E
F
Up-regulated Proteins
Down-regulated Proteins
Down-regulated Proteins

## Slide 2
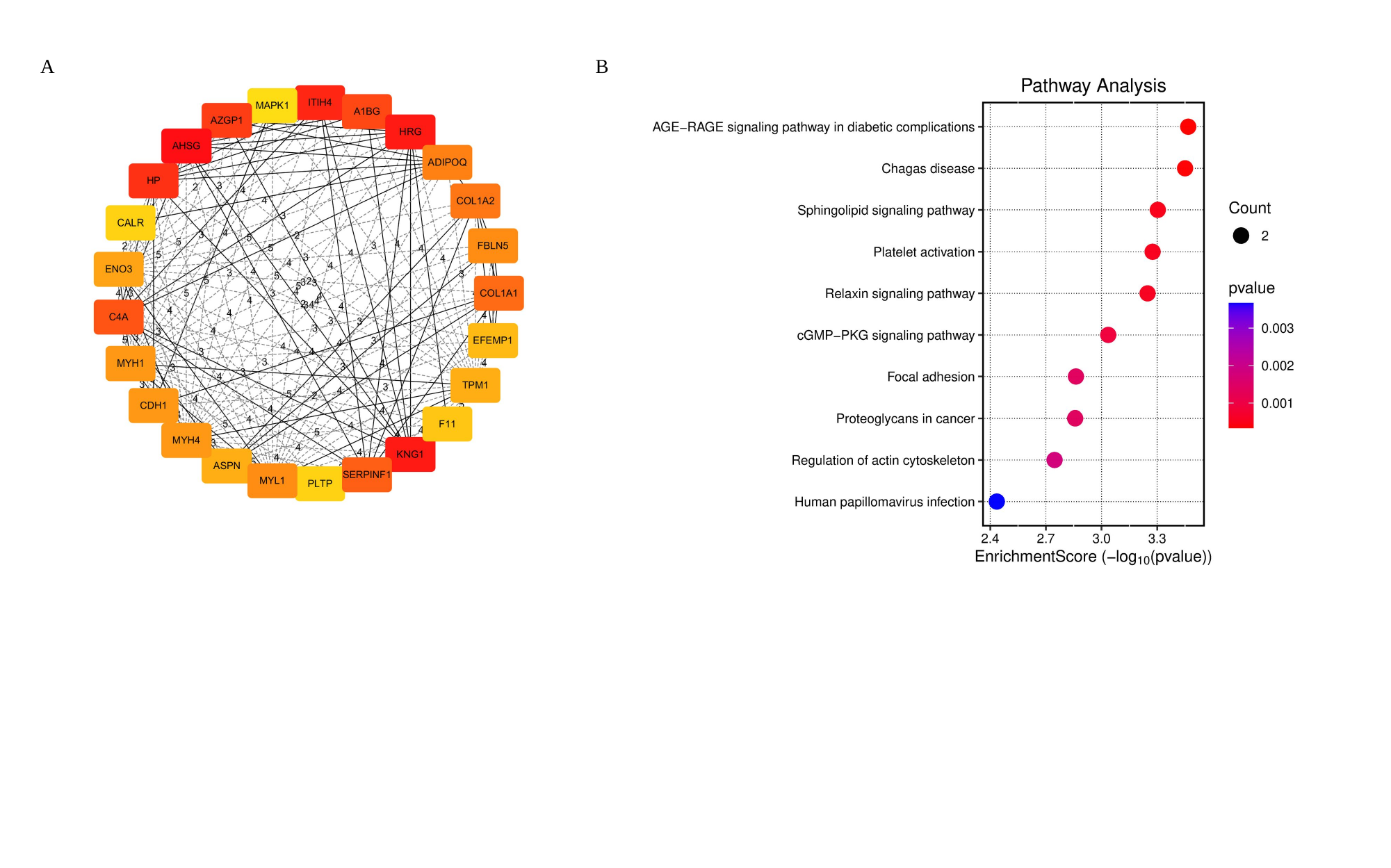

A
B

## Slide 3
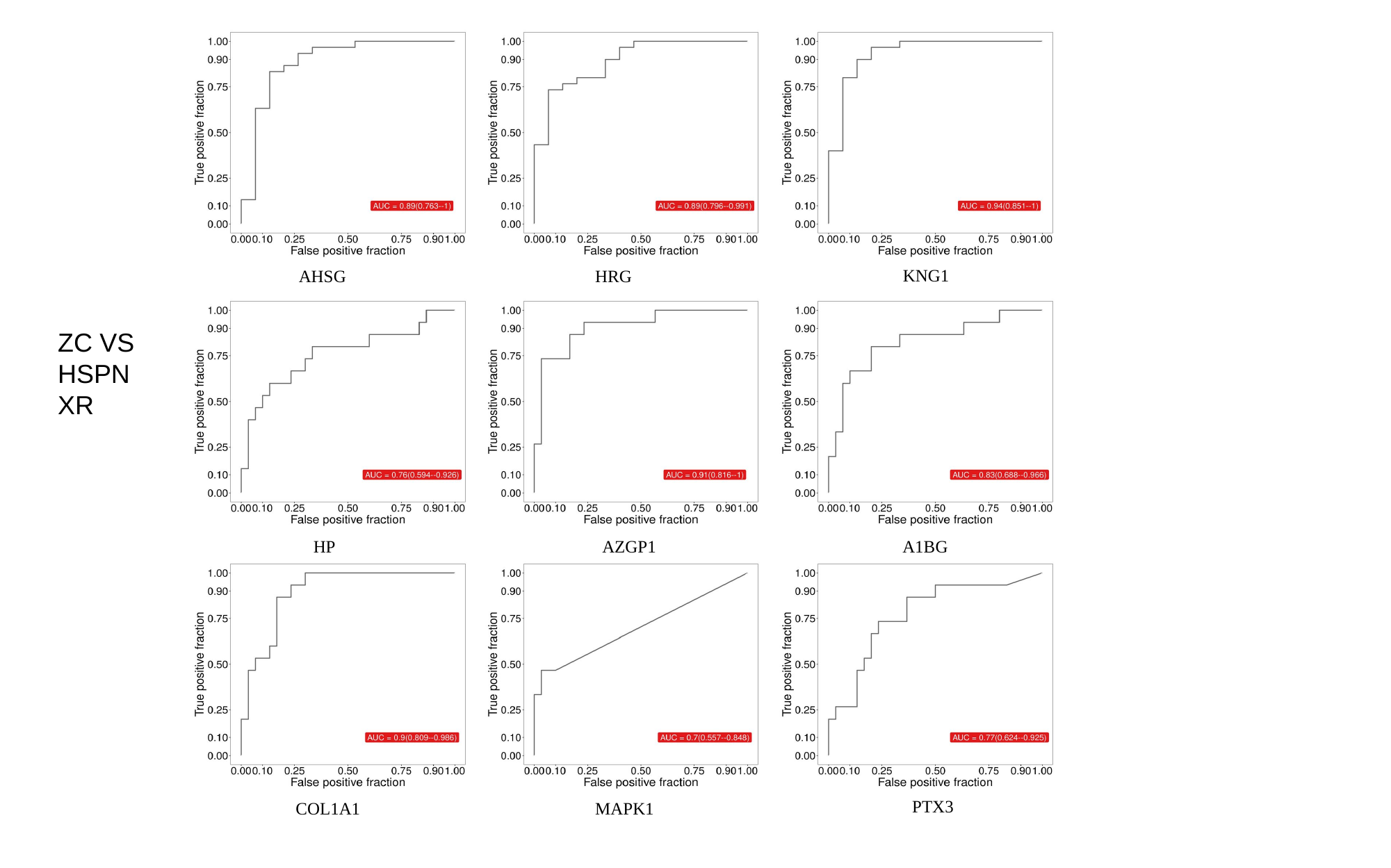

KNG1
AHSG
HRG
HP
AZGP1
A1BG
PTX3
COL1A1
MAPK1
ZC VS
HSPNXR

## Slide 4
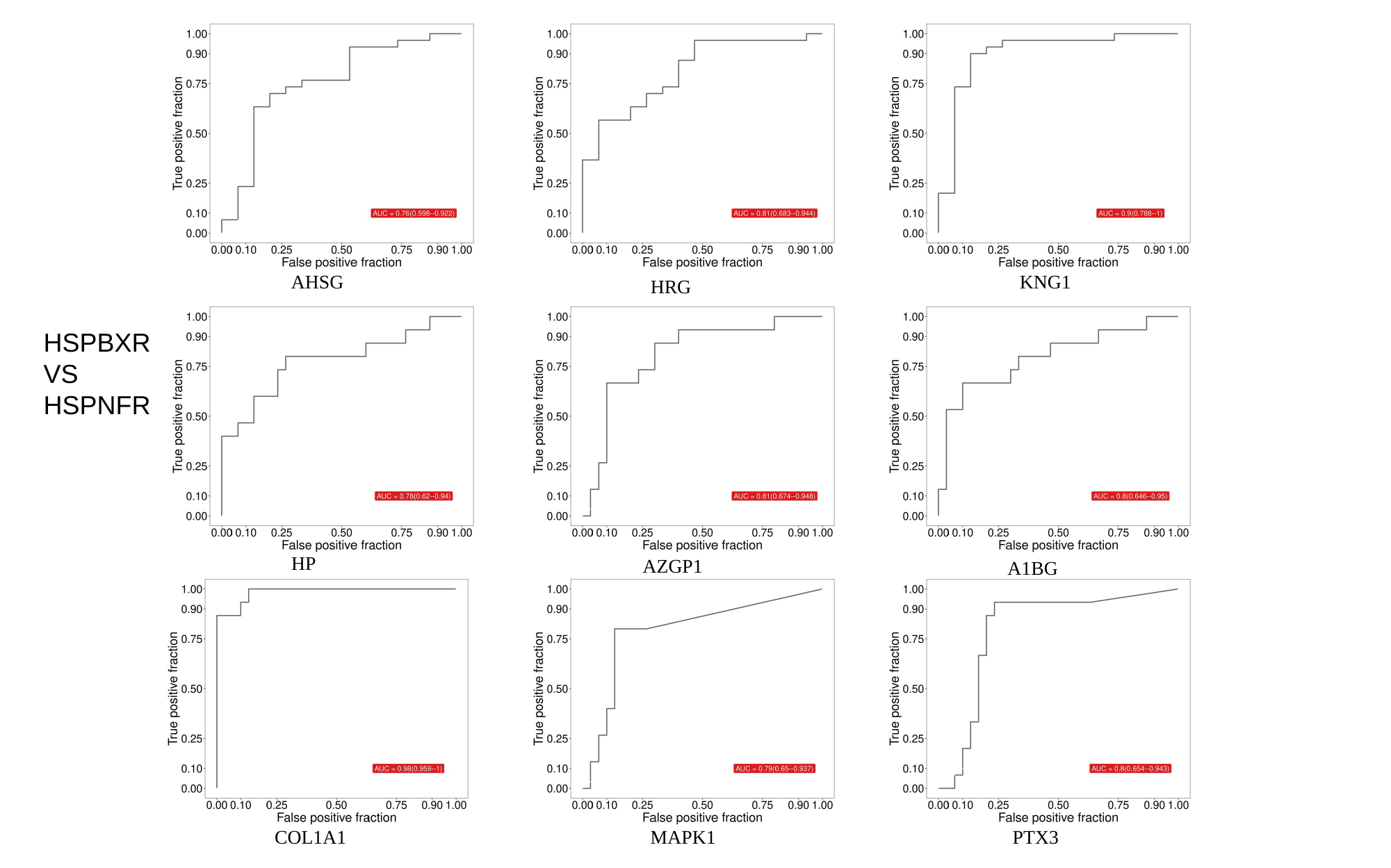

AHSG
KNG1
HRG
HSPBXR VS
HSPNFR
HP
AZGP1
A1BG
PTX3
COL1A1
MAPK1

## Slide 5
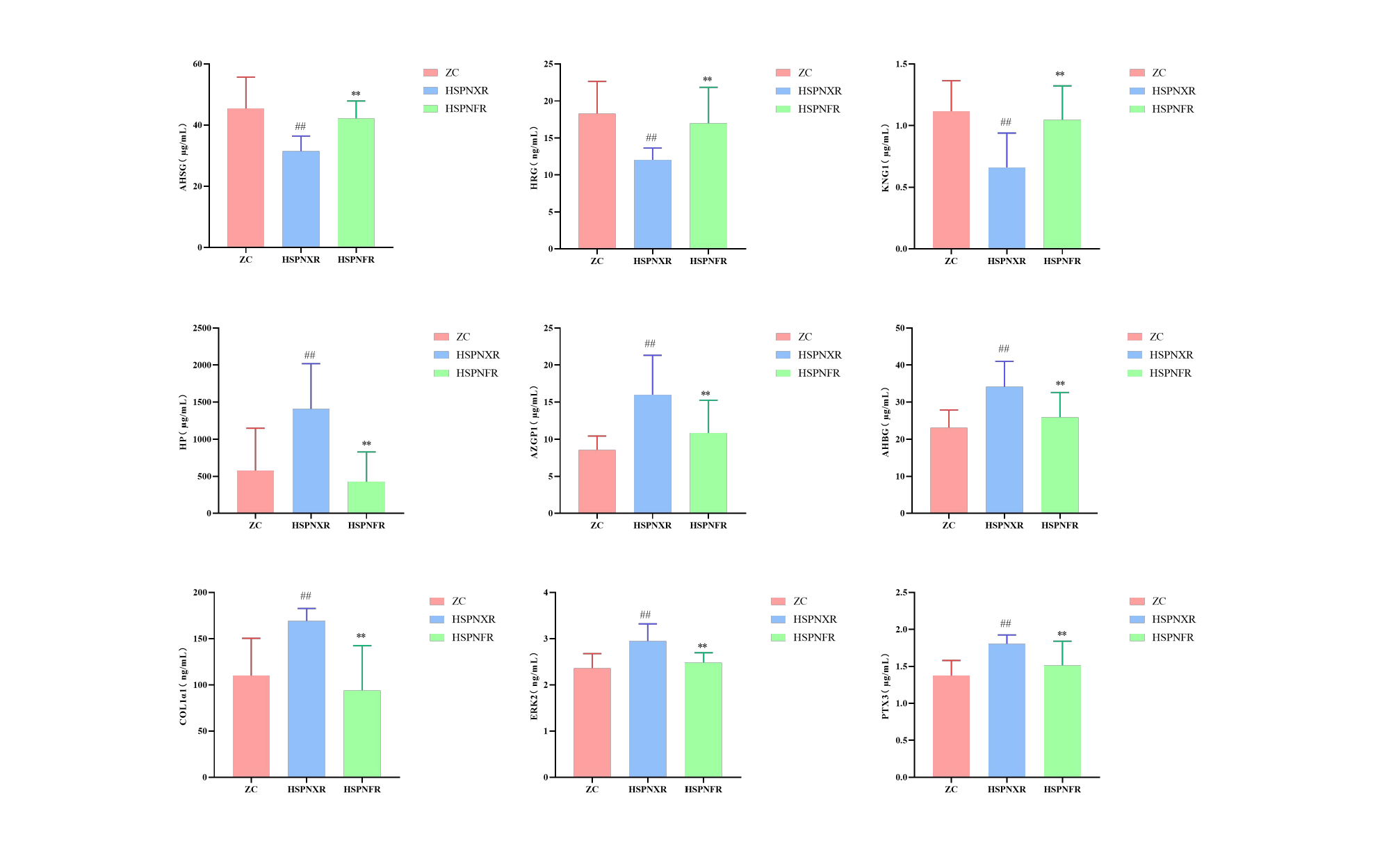

## Slide 6
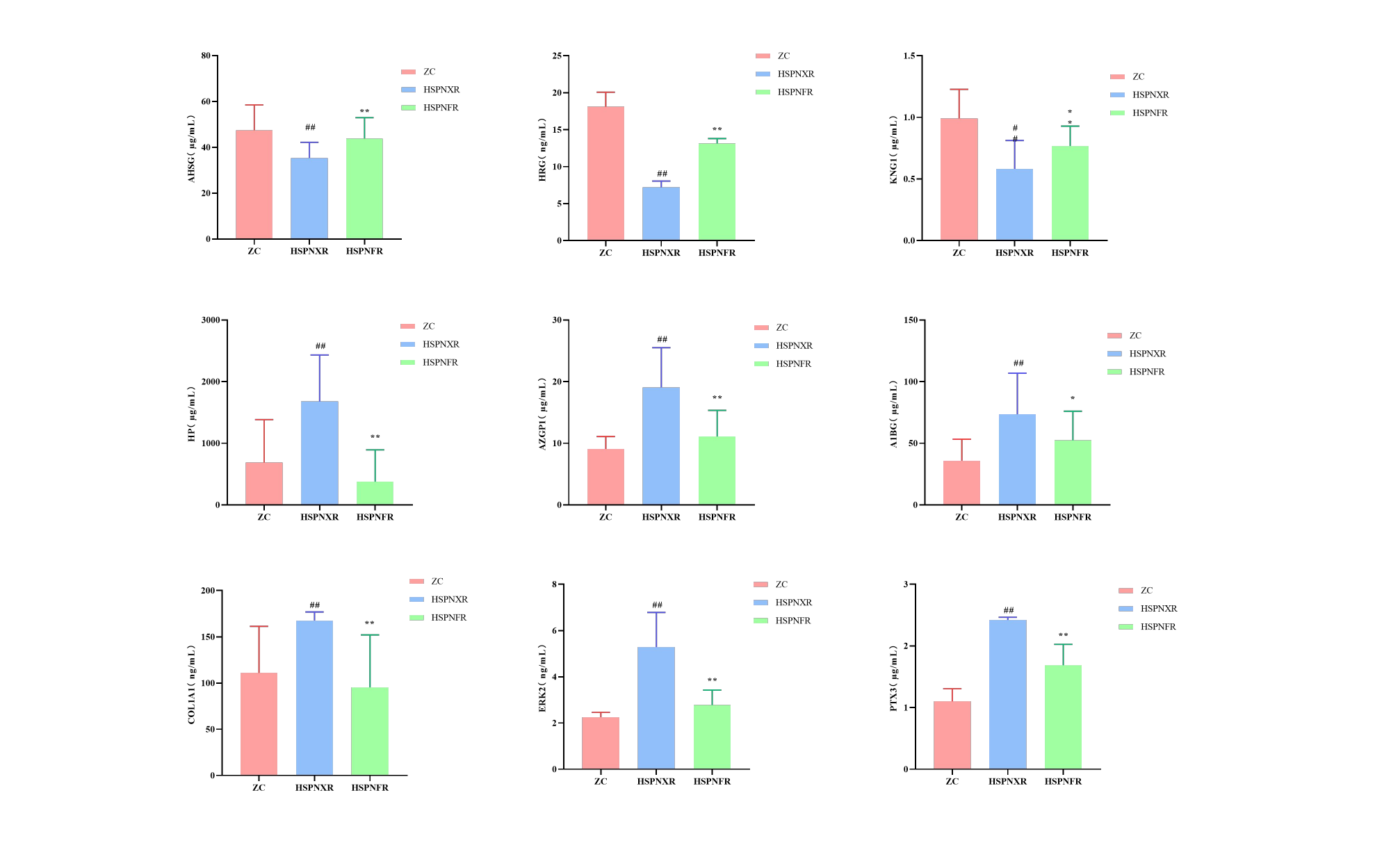

Supplement: Supplementary file 3 [file Supplementaryfile2.pptx]
