# Supplementary material for: The biological basis of Blood-Heat syndrome in children with Henoch-Schonlein purpura nephritis: a multidimensional analysis based on clinical proteomics and an animal model
Source: Front Pharmacol. 2026 Apr 10;17:1778919. doi: 10.3389/fphar.2026.1778919 (PMC13105992; doi:10.3389/fphar.2026.1778919)
Supplement: Supplementary file 4 [file Supplementaryfile1.pptx]

## Slide 1
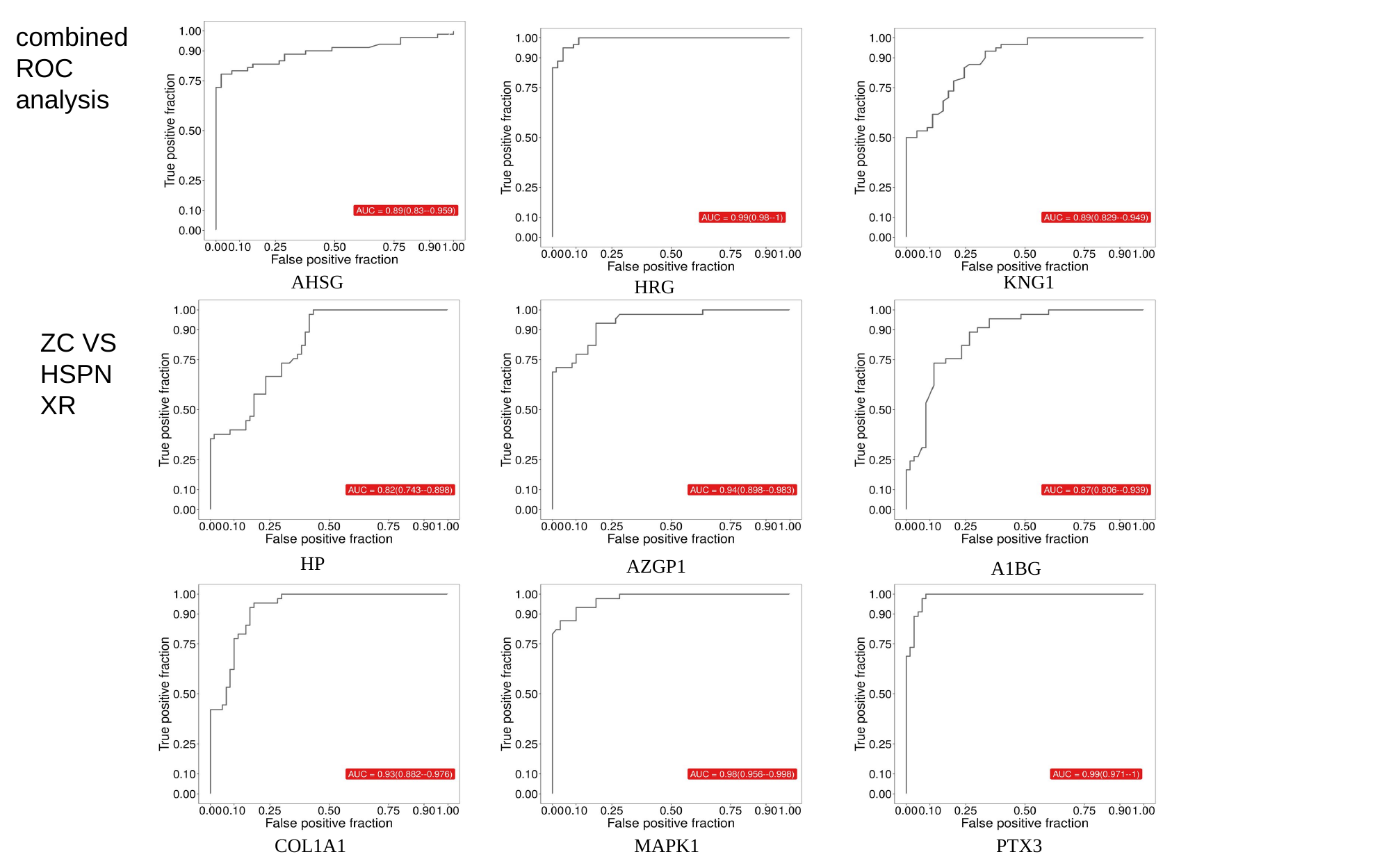

combined ROC analysis
AHSG
KNG1
HRG
ZC VS
HSPNXR
HP
AZGP1
A1BG
PTX3
COL1A1
MAPK1

## Slide 2
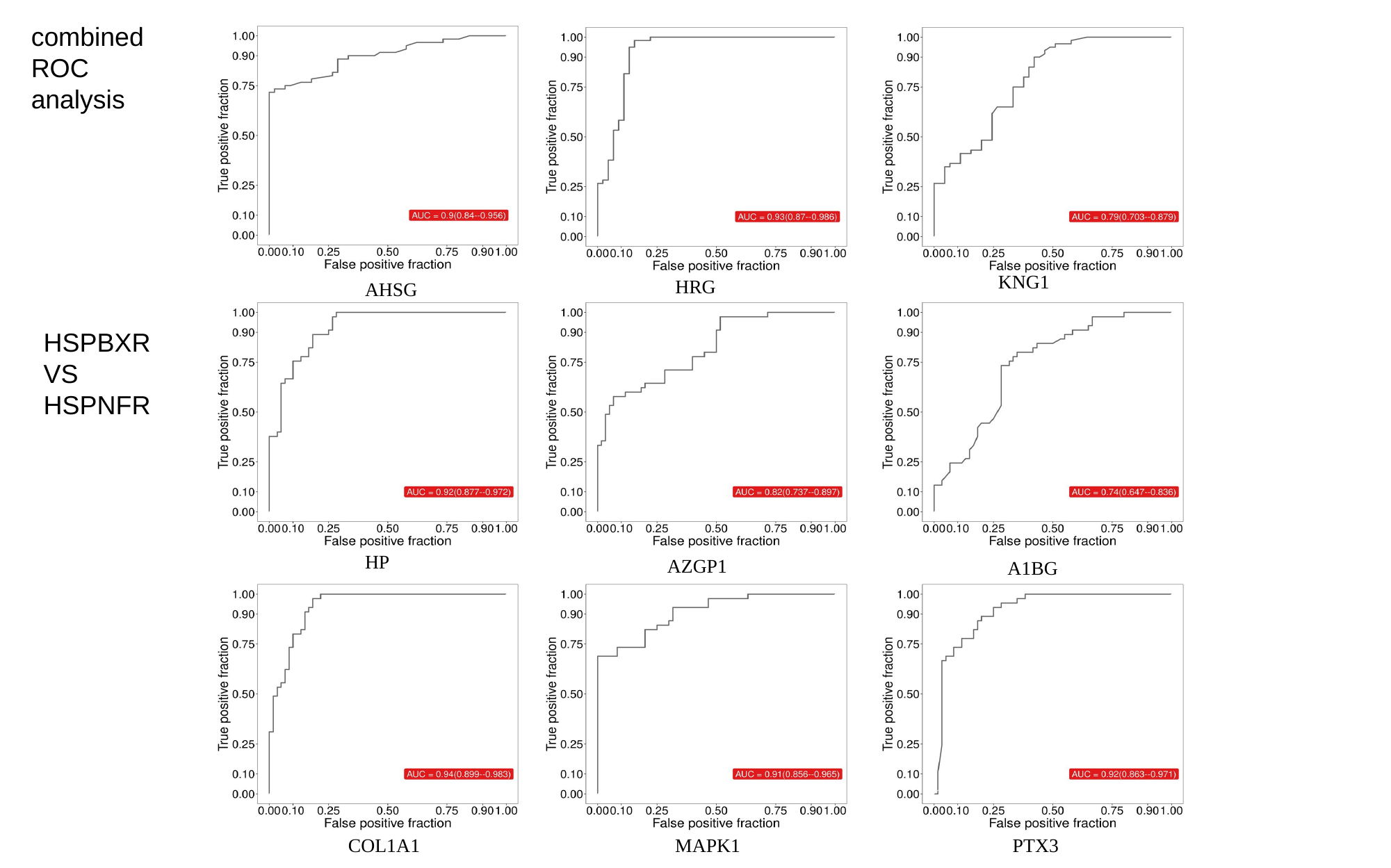

combined ROC analysis
KNG1
HRG
AHSG
HSPBXR VS
HSPNFR
HP
AZGP1
A1BG
PTX3
COL1A1
MAPK1
