# Supplementary material for: The biological basis of Blood-Heat syndrome in children with Henoch-Schonlein purpura nephritis: a multidimensional analysis based on clinical proteomics and an animal model
Source: Front Pharmacol. 2026 Apr 10;17:1778919. doi: 10.3389/fphar.2026.1778919 (PMC13105992; doi:10.3389/fphar.2026.1778919)
Supplement: Supplementary file 5 [file Supplementaryfile4.docx]

**Supplementary Table 1. Comparison of Baseline Characteristics Between the ZC Group and the HSPNXR Group in the Discovery Cohort**

| Variable | ZC Group (n=30) | HSPNXR Group (n=15) | *P* | Statistic |
| --- | --- | --- | --- | --- |
| Age (years) | 8.87±1.78 | 8.93±2.28 | 0.915 | -0.108 |
| Gender |  |  |  |  |
| Boys | 20(66.6%) | 6（40%） | 0.088 | 2.915 |
| Girls | 10(33.3%) | 9（60%） |  |  |
| Body Weight（kg） | 34.17±8.57 | 33.46±8.52 | 0.796 | 0.26 |
| Height（cm） | 141.97±13.9 | 141.00±11.18 | 0.816 | 0.234 |

**Supplementary Table 2. Comparison of Baseline Characteristics Between the ZC Group and the HSPNFR Group in the Discovery Cohort**

| Variable | ZC Group (n=30) | HSPNFR Group (n=30) | *P* | Statistic |
| --- | --- | --- | --- | --- |
| Age (years) | 8.87±1.78 | 8.97±3.33 | 0.885 | -0.145 |
| Gender |  |  |  |  |
| Boys | 20(66.6%) | 16（53.3%） | 0.292 | 1.111 |
| Girls | 10(33.3%) | 14（46.7%） |  |  |
| Body Weight（kg） | 34.17±8.57 | 30.38±10.57 | 0.133 | 1.524 |
| Height（cm） | 141.97±3.9 | 135.70±19.63 | 0.159 | 1.427 |

**Supplementary Table 3. Comparison of Baseline Characteristics Between the HSPNXR Group and the HSPNFR Group in the Discovery Cohort**

| Variable | HSPNXR Group  (n=15) | HSPNFR Group  (n=30) | *P* | Statistic |
| --- | --- | --- | --- | --- |
| Age (years) | 8.93±2.28 | 8.97±3.33 | 0.972 | -0.035 |
| Gender |  |  |  |  |
| Boys | 6（40%） | 16（53.3%） | 0.399 | 0.711 |
| Girls | 9（60%） | 14（46.7%） |  |  |
| Body Weight（kg） | 141.00±11.18 | 135.70±19.63 | 0.256 | 1.152 |
| Height（cm） | 33.46±8.52 | 30.38±10.57 | 0.333 | 0.979 |
| Variable | 15（11，45） | 30（20，47.25） | 0.196 | -1.293 |

**Supplementary Table 4. Comparison of Baseline Characteristics Between the ZC Group and the HSPNXR Group in the Validation Cohort**

| Variable | ZC Group(n=30) | HSPNXR Group (n=30) | *P* | Statistic |
| --- | --- | --- | --- | --- |
| Age (years) | 8.87±1.78 | 9.40±2.33 | 0.323 | -0.998 |
| Gender |  |  |  |  |
| Boys | 20（66.6%） | 18（60%） | 0.592 | 0.287 |
| Girls | 10（33.3%） | 12（40%） |  |  |
| Body Weight（kg） | 34.17±8.57 | 30.70±8.14 | 0.114 | 1.606 |
| Height（cm） | 141.97±13.9 | 141.57±14.57 | 0.914 | 0.109 |

**Supplementary Table 4. Comparison of Baseline Characteristics Between the ZC Group and the HSPNFR Group in the Validation Cohort**

| Variable | ZC Group(n=30) | HSPNFR Group(n=30) | *P* | Statistic |
| --- | --- | --- | --- | --- |
| Age (years) | 8.87±1.78 | 10.2±3.32 | 0.059 | -1.942 |
| Gender |  |  |  |  |
| Boys | 20（66.6%） | 19（63.3%） | 0.787 | 0.073 |
| Girls | 10（33.3%） | 11（36.7%） |  |  |
| Body Weight（kg） | 34.17±8.57 | 34.80±9.44 | 0.786 | -0.273 |
| Height（cm） | 141.97±13.90 | 145.13±20.73 | 0.49 | -0.695 |

**Supplementary Table 6. Comparison of Baseline Characteristics Between the HSPNXR Group and the HSPNFR Group in the Validation Cohort**

| Variable | HSPNXR Group(n=30) | HSPNFR Group(n=30) | *P* | Statistic |
| --- | --- | --- | --- | --- |
| Age (years) | 9.70±2.52 | 10.93±3.38 | 0.262 | -1.133 |
| Gender |  |  |  |  |
| Boys | 18（60%） | 19（63.3%） | 0.791 | 0.071 |
| Girls | 12（40%） | 11（36.7%） |  |  |
| Body Weight（kg） | 30.70±8.14 | 35.10±9.43 | 0.077 | -1.801 |
| Height（cm） | 141.57±14.57 | 145.47±20.47 | 0.444 | -0.771 |
| Variable | 30（18，60） | 30（20,90） | 0.518 | -0.647 |
